# A Review and Research Proposal on Pioneering Sustainable Unmanned Aerial Vehicles (UAVs) with Kenaf Fibre Biocomposites for Structural and Electronic Integration

**DOI:** 10.3390/ma19163451

**Published:** 2026-08-14

**Authors:** Thinesh Sharma Balakrishnan, Khalina Abdan, Krzysztof Nozdrzykowski, Rafał Grzejda, Mohd Radzi Ali, Suhas Yeshwant Nayak, Anand Pai

**Affiliations:** 1 Laboratory of Biocomposite Technology, Institute of Tropical Forestry and Forest Products (INTROP), Universiti Putra Malaysia, Serdang 43400, Selangor, Malaysia; thineshsharma@upm.edu.my (T.S.B.); mohdradzi@upm.edu.my (M.R.A.); 2 Department of Biological and Agricultural Engineering, Faculty of Engineering, Universiti Putra Malaysia, Serdang 43400, Selangor, Malaysia; 3 Faculty of Marine Engineering, Maritime University of Szczecin, 70-500 Szczecin, Poland; k.nozdrzykowski@pm.szczecin.pl; 4 Faculty of Mechanical Engineering and Mechatronics, West Pomeranian University of Technology in Szczecin, 70-310 Szczecin, Poland; 5 Manipal Institute of Technology, Manipal Academy of Higher Education, Manipal 576104, India; suhas.nayak@manipal.edu (S.Y.N.); anand.pai@manipal.edu (A.P.)

**Keywords:** kenaf fibres, natural fibre composites, additive manufacturing, unmanned aerial vehicles, aerospace materials, sustainability

## Abstract

**Highlights:**

- Kenaf fibre biocomposites are positioned as sustainable alternatives to carbon and glass fibres for UAV applications, offering low density, renewable sourcing and significant carbon footprint reduction.
- The review systematically covers kenaf integration across UAV structures, electronics, and additive manufacturing, including KNFRP panels, pellets, 3D-printing filaments, pressed paper, foams and biofilms.
- Kenaf-based hybrid composites demonstrate enhanced mechanical, thermal and impact performance, enabling their use in semi-structural and selected load-bearing UAV components.
- Kenaf-based materials show strong potential for UAV electronics, providing electrical insulation, vibration damping, acoustic control, and thermal management for PCBs and sensor systems.
- Key challenges, such as moisture sensitivity, thermal stability, recyclability, and supply-chain scalability, are critically discussed, with mitigation strategies and future research directions outlined to enable practical UAV adoption.

**Abstract:**

Unmanned aerial vehicles (UAVs) are experiencing rapid growth across diverse sectors, creating an increasing demand for lightweight, high-performance and environmentally sustainable materials. Conventional drone materials offer excellent mechanical properties but pose environmental concerns due to their high carbon footprint, energy-intensive production and limited biodegradability. Kenaf fibre, a renewable natural fibre, presents a promising alternative owing to its low density, high specific strength, cost-effectiveness and eco-friendly characteristics. This review and research proposal explores the current and potential applications of kenaf-based materials in drone manufacturing, including kenaf fibre-reinforced biocomposites, pressed paper, composite pellets and 3D printing filaments for structural, functional and electrical housing components. Kenaf-based materials have demonstrated mechanical strengths approaching 300 MPa, dielectric constants of approximately 2.5 and electrical breakdown strengths exceeding 150 kV/mm, highlighting their potential for lightweight UAV structures and electronic insulation applications. The proposed research focuses on optimising kenaf fibre treatment, fibre–matrix compatibility, hybrid reinforcement strategies and additive manufacturing parameters to develop lightweight, durable and multifunctional kenaf-based UAV components. The framework aims to establish a systematic pathway for the development and validation of kenaf-based materials for next-generation sustainable UAVs.

## 1. Introduction

Unmanned aerial vehicles (UAVs), commonly referred to as drones, have gained widespread attention across various sectors due to their versatility and efficiency. Applications of UAVs span military reconnaissance [1], precision agriculture [2], disaster management [3], search and rescue [4] and commercial deliveries. UAVs are broadly categorised into fixed-wing, rotary-wing (e.g., quadcopters) and hybrid configurations, with each type tailored for specific operational needs [5]. For example, fixed-wing UAVs excel in long-duration flights, while quadcopters offer superior manoeuvrability and are ideal for surveillance and close-range applications. Fibres played a significant role in the development of UAVs, as they are lightweight, possess excellent mechanical properties and corrosion resistance [6]. Carbon fibre was frequently employed in primary structural components such as the fuselage, wings and propellers, where its high stiffness-to-weight ratio contributed to enhanced aerodynamic efficiency [7]. Glass fibre, while economical, provided sufficient impact resistance and was typically used in secondary structures [8]. Aramid fibres, including Kevlar, were chosen for their toughness and were applied in areas requiring vibration damping or localised impact protection. The integration of these fibre-reinforced composites improved structural reliability, supported greater payload capacity and contributed to more efficient flight dynamics.

Vedrtnam et al. [9] investigated the use of 3D-printed glass fibre- and carbon fibre-reinforced Onyx composites for drone airframe applications. Their numerical and experimental findings showed that the Onyx + carbon fibre composite achieved a Young’s modulus of 16.9 GPa and an ultimate tensile strength of 418 MPa, with improvements of 316.6% in flexural strength and 1344% in tensile strength compared with neat Onyx, while finite element analysis (FEA) [10] further confirmed that interlaminar stresses were the dominant failure mechanism under tensile loading, supporting its suitability for high-performance UAV frame fabrication.

In pursuit of more sustainable solutions, natural fibres have emerged as promising alternatives, offering reduced environmental impact while maintaining acceptable performance for specific UAV applications [6]. Previous research has explored the use of bio composites in UAV applications, showcasing their potential to balance performance and sustainability. Lopena and Millare [11] evaluated the potential of salago fibre composite as an alternative material for drone airframes and compared its performance with conventional glass fibre composites. Their findings showed that although glass fibre exhibited higher flexural and impact strength, the salago fibre composite demonstrated a lower density of 1.19 g/cm^3^ (about 4.8% lighter) and a higher heat deflection temperature of 58.6 °C (7.9% higher), while also successfully functioning as a drone frame that withstood repeated 2 m drop tests, highlighting its potential as a lightweight and sustainable UAV airframe material.

Prakash et al. [12] investigated green pea pod lignin macromolecules, industrial hemp fibre and polyester resin for defence and surveillance drones. Their study demonstrated favourable wear rates (0.103 mm^3^/Nm) and low coefficients of friction (0.35) at 4.0% lignin volume, along with promising fatigue life (36,972 cycles at 25% of the ultimate tensile strength) and low flame propagation speeds (11.90 mm/min).

Lefebvre et al. [13] examined flax/Elium composites for autonomous long-range drones and compared them to glass and carbon fibre Elium composites. FEA revealed lower resistance to compression for flax/Elium, with failure occurring prematurely compared to glass fibre structures, indicating the need for further optimisation.

Dev et al. [14] investigated the mechanical and moisture absorption behaviour of sugarcane bagasse fibre-reinforced epoxy composites for drone frame development. The researchers found that neat epoxy exhibited superior overall properties, while composites containing 4 wt.% fibre showed improved tensile and flexural strength compared with lower fibre loadings. Their study also showed that alkali treatment reduced fibre weight and moisture absorption, highlighting the importance of optimising fibre treatment, fibre length, and loading to enhance the performance of sugarcane bagasse/epoxy composites.

Singsang et al. [15] reported that hemp fibre-reinforced polypropylene (PP) composites with 45 wt.% fibre content achieved the highest Young’s modulus of 1169.40 MPa, approximately twice that of neat PP, indicating improved stiffness for drone frame applications. However, tensile strength decreased with increasing fibre content and the strain at break dropped to 3.97–8.42% compared with 28.31% for pure PP, showing reduced ductility. Despite this, the 45 wt.% composite exhibited impact strength close to PP and withstood a simulated maximum stress of 1.637 MPa under a 0.64 N load, supporting its feasibility as an alternative lightweight material for drone structures.

Johny et al. [16] developed a coconut coir-based porous carbon/magnetic iron composite as a sustainable electromagnetic shielding material for UAV applications. The composite exhibited strong electromagnetic wave attenuation, achieving reflection loss values of −26.41 dB at 2 mm thickness and −36.68 dB at 4 mm thickness, indicating excellent shielding performance across a broad frequency range. These findings demonstrate the potential of composites of this type as a lightweight, cost-effective and eco-friendly material for improving electromagnetic compatibility in UAV systems.

Another study by Prakash et al. [17] demonstrated the development of a lightweight epoxy-based biocomposite (BC) [18,19] for morphing wing and UAV applications using a 3D-printed lignin-acrylonitrile butadiene styrene core reinforced with hemp and aluminium-coated glass fibre epoxy skin. Their optimum composite (E2) achieved a tensile strength of 136 MPa, flexural strength of 168 MPa, compression strength of 155 MPa, Izod impact strength of 4.82 kJ/m^2^ and interlaminar shear strength of 21 MPa. In addition, the E2 composite absorbed 20.6 J during impact testing, demonstrated fatigue life cycles of 33,709; 25,781 and 19,633 at 50%, 70% and 90% of the ultimate tensile strength, respectively, and showed a fracture toughness (K_1_c) of 32.5 $\mathrm{MPa}\sqrt{m}$ with an energy release rate (G_1_c) of 0.76 MJ/m^2^.

Senan et al. [20] applied the analytic hierarchy process to evaluate polylactic acid (PLA) reinforced with 6.3% jute (PLA/Jute), PP reinforced with 30% harakeke (PP/Harakeke) and PP reinforced with 30% hemp (PP/Hemp) for drone frame structures. The analysis identified performance (0.337) as the most important criterion, followed by environmental impact (0.311), cost (0.193), weight (0.086) and size (0.073). Among the materials assessed, PLA/Jute achieved the highest overall score of 0.449, with 0.417 for tensile strength and 0.478 for modulus, outperforming PP/Harakeke and PP/Hemp as the preferred natural fibre composite filament for drone structural applications.

**Figure 1:**
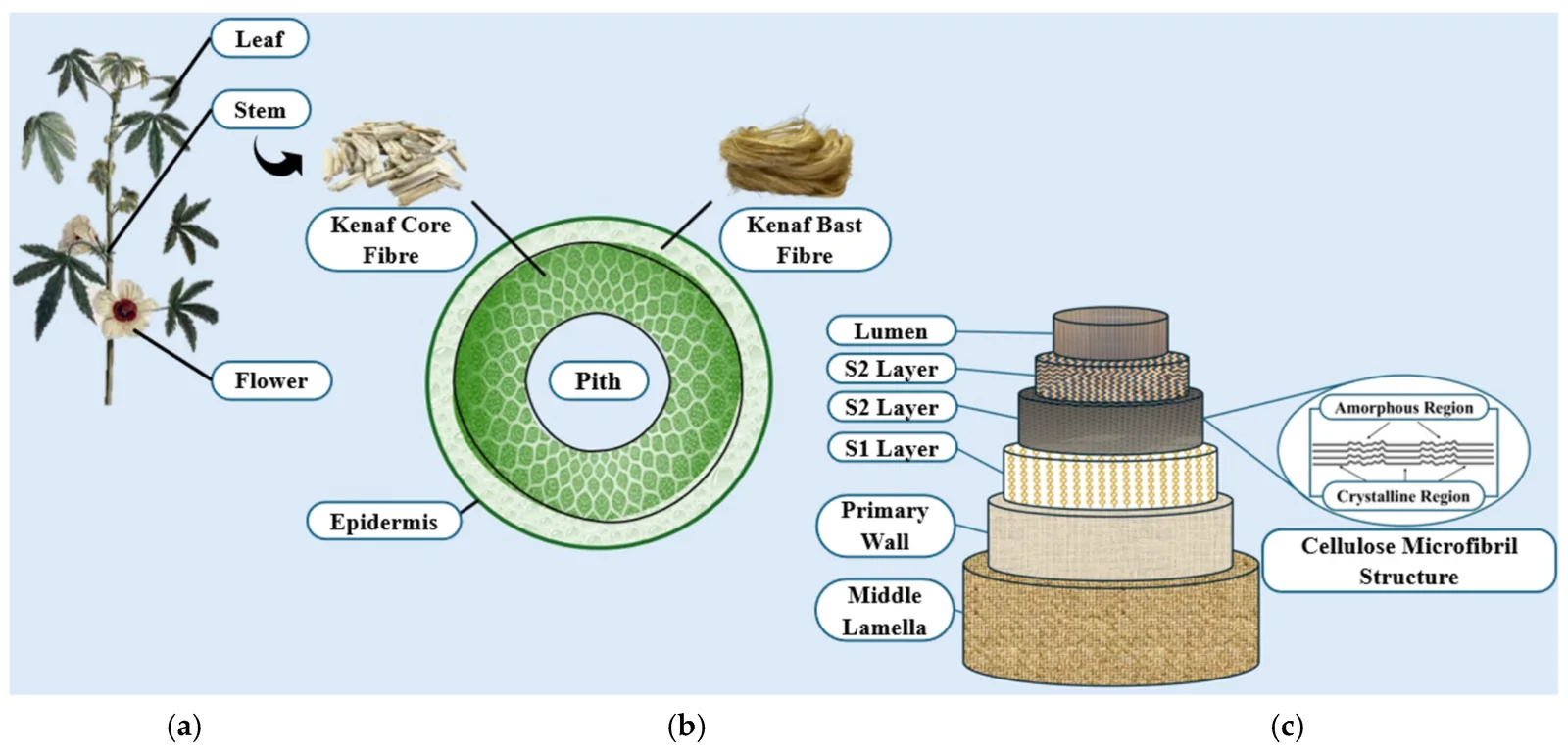
Features of KFs: (**a**) kenaf plant; (**b**) anatomy of bast and core fibres in kenaf stem; (**c**) microstructure of kenaf bast fibre.

Kenaf fibres (KFs), locally sourced from Malaysia, present a promising sustainable alternative for UAV manufacturing [21]. While studies highlight the viability of BCs for UAVs, a significant gap remains in exploring KFs. Despite their promising properties, including lightweight, high tensile strength and environmental sustainability, KFs have yet to be fully integrated into UAV design, presenting an opportunity for further research and innovation. As shown in Figure 1, kenaf consists of bast fibres, which are long, strong and flexible, making them ideal for high-strength BCs, textiles, premium paper and insulation materials. On the other hand, kenaf core fibres are lightweight, spongy and highly absorbent, suitable for construction materials, composite fillers [22], animal bedding, oil spill absorbents and low-density paper products [23].

Chandran et al. [24] investigated the use of kenaf fibre-reinforced composite (KFRC) for quadcopter arm structures using FEA, focusing on fibre orientation and cross-sectional design. Their results showed that the circular hollow tube provided the highest stiffness with the lowest deflection, while the 0°/30°/45°/30°/0° fibre orientation achieved the highest maximum stress (0.3427 MPa) and the 0°/45°/0°/45°/0° configuration showed the lowest displacement. Arowolo et al. [25] investigated flax, vegetal-technic, woodforce, and hybrid flax/vegetal-technic fibre-reinforced PP composites using micromechanics and multiscale modelling for quadcopter frame applications. Their analysis evaluated fibre orientations of 0°, 45°, 90° and random, and established a material model for a flax–PP composite with 50% fibre volume fraction, showing good agreement between FEA, analytical and reported experimental results. The quadcopter frame simulation further demonstrated that the plant fibre composites were able to withstand thermomechanical loading under extreme temperatures of −20 °C and 55 °C, highlighting their suitability for sustainable UAV structural applications.

Kenaf core has been explored as an alternative fine aggregate in construction materials, such as concrete [26,27] and kenaf core quarry dust bricks. A study by Hassan et al. [28] found that increasing kenaf core content in the bricks reduced compressive strength and density while increasing porosity. Additionally, the thermal insulation also improved as the thermal conductivity decreased from 0.63 W/mK to 0.42 W/mK when kenaf core content increased from 5% to 25%. With attributes such as high tensile strength, lightweight properties and eco-friendliness, KFs are well-positioned to replace traditional UAV materials like aluminium, carbon and glass fibre. These fibres are particularly attractive in reducing the environmental footprint of UAV production while maintaining structural integrity [29]. According to Abdelrhman et al. [30], in Malaysia, kenaf cultivation for industrial applications helps to reduce carbon emissions by absorbing about 180,000 tonnes of CO_2_ per hectare each year. The overall carbon footprint of growing kenaf is low, at 0.750 tonnes of CO_2_ per tonne of kenaf produced, making it an environmentally friendly option.

Globally, the use of natural fibre composites has expanded across various industries. Table 1 shows the integration of natural fibre BCs into various industries worldwide, highlighting the urgent need for sustainable alternatives to conventional synthetic materials. Companies such as Bcomp (Fribourg, Switzerland), Greenboats (Bremen, Germany) and FlexForm Technologies (Elkhart, IN, USA) have successfully commercialised composites made from flax, hemp, jute, cork and wood fibres across sectors ranging from automotive and aerospace to furniture and construction. These BCs offer properties such as lightweight strength, thermal stability, acoustic insulation and eco-friendliness, reducing carbon footprints while meeting demanding industrial standards. Despite these advancements, KF, known for its high specific strength, low density and carbon sequestration potential, remains underutilised, particularly in high-value industries like aerospace and UAV manufacturing. Given the proven success of other natural fibres, there is a strong case for the wider integration of kenaf into composite markets, leveraging Malaysia’s established kenaf supply chain to develop sustainable, lightweight and high-performance components for emerging applications such as UAVs.

This review systematically explores the integration of KFs into the UAV materials industry. The growing demand for sustainable, high-performance materials in UAV applications highlights the urgent need for comprehensive studies on alternative solutions like KF. Despite significant advancements in BCs, the integration of KF into UAV design remains underexplored, particularly in addressing the unique challenges posed by structural and electronic components. This review aims to fill this gap by systematically exploring the potential of KF and kenaf natural fibre-reinforced polymers (KNFRPs) in UAV applications, focusing on the latest research and innovations from the past five years. It delves into two critical UAV components: structures, which demand lightweight yet durable materials for enhanced aerodynamics and resilience, and electronics, where KNFRPs can provide sustainable substrates for printed circuit boards, insulation panels and other components. By highlighting how KNFRPs can seamlessly integrate with current manufacturing techniques and technologies, this review seeks to inspire further innovation and provide a roadmap for future research in sustainable UAV engineering.

## 2. Potential Applications of Kenaf in UAVs

In this section, the diverse applications of KFs and KFRCs in UAV systems are explored, particularly in structural components where their lightweight and sustainable properties are advantageous. It also discusses the use of various moulding techniques to shape kenaf-based pellets into complex geometry parts and explores the emerging potential of 3D printing for creating customised UAV components with kenaf composites. Additionally, this section highlights the application of KNFRP boards, kenaf pressed paper (KPP) and bioplastic films as eco-friendly alternatives for UAV electronics such as printed circuit boards (PCBs), sensor attachments and electronic insulators, contributing to sustainable aerospace innovations.

### 2.1. KNFRP for UAV Structures

KNFRP is a BC material in which KFs are embedded in a polymer matrix to create a lightweight, sustainable alternative to synthetic fibre composites. KFs, derived from the *Hibiscus cannabinus* plant, offer good specific strength, low density and biodegradability. Various polymer matrices can be used to bind KFs, including thermosetting resins like epoxy, polyester [46] and vinyl ester, which provide good mechanical properties and durability, as well as thermoplastics like PP and PLA for recyclable and biodegradable composites. The manufacturing methods for UAV composites using KNFRP include hand lay-up, which is cost-effective but labour-intensive; vacuum bagging, which enhances fibre consolidation and reduces voids; resin transfer moulding for high-quality, complex shapes; and compression moulding, which allows high-volume production with consistent properties.

Current UAVs primarily use carbon fibre-reinforced polymer (CFRP) for high-strength airframes, glass fibre-reinforced polymer (GFRP) for secondary structures, aluminium for load-bearing components, aramid fibre-reinforced polymer (AFRP) for impact-resistant parts like rotor blades and plastics for lightweight housing, while KNFRP is emerging as an eco-friendly alternative for non-critical structures. Fernandez et al. [47] analysed the structural strength of UAV tube and connector parts using carbon fibre and aluminium 6061 alloy through finite element simulations in stable flight conditions. The maximum stress values for tubes were 15.71 MPa (carbon fibre) and 15.64 MPa (aluminium 6061), both well below their respective yield stresses (551 MPa and 55.1 MPa). The safety factor for tubes was 35 (carbon fibre) and 3.5 (aluminium 6061), with maximum displacement values of 0.056 mm and 0.812 mm, respectively. For connectors, maximum stress values were 4.266 MPa (carbon fibre) and 4.139 MPa (aluminium 6061), with safety factors of 130 and 3.5, and maximum displacements of 3.734 × 10^−5^ mm and 5.416 × 10^−4^ mm. The results confirm that carbon fibre provides superior structural strength and lower displacement when compared to aluminium.

Dhinnesh S et al. [48] compared aluminium, CFRP and GFRP for UAV propellers using ANSYS version 2023 R1. CFRP exhibited the highest von Mises stress (0.060 MPa) and lowest deformation (0.000655 mm), making it the strongest and stiffest option, while aluminium had the lowest stress (0.0359 MPa) and highest strain (6.66 × 10^7^), making it more lightweight but less stiff, and GFRP provided a cost-effective balance with moderate stress (0.0519 MPa) and deformation (0.00144 mm).

Plastics exhibit low strength but high elongation, making them useful for lightweight, non-structural UAV components like fairings, covers and housings where flexibility is required. Aluminium, with its moderate strength and good ductility, is ideal for UAV frames and landing gear, as it can withstand moderate impacts while remaining lightweight. GFRP is stronger than aluminium but more brittle, making it a good choice for secondary structural parts like wings, control surfaces and radar domes, where weight savings are important, but extreme loads are not a primary concern. CFRP has the highest strength and stiffness but is brittle, making it perfect for primary UAV structures like fuselages, wing spars and high-performance airframes, particularly for high-speed, long-endurance or high-altitude UAVs, where rigidity is critical [49]. AFRP balances strength and toughness, making it well-suited for impact-resistant applications such as UAV propellers, rotor blades and protective shielding, especially in military or rugged UAVs that operate in unpredictable conditions. Sonmez et al. [50] developed and tested thermoplastic composites reinforced with SiO_2_-modified aramid fibres, demonstrating 20% higher tensile strength, 36–52% higher Young’s modulus, 26–33% higher flexural strength and 30–47% higher flexural modulus, along with 20–40% improved impact resistance compared to control samples. The composites were successfully vacuum-thermoformed into UAV covers, with 3D-printed moulds enabling easier demoulding and added structural rigidity, and the covers withstood real-world flight tests under standard outdoor conditions.

**Figure 2:**
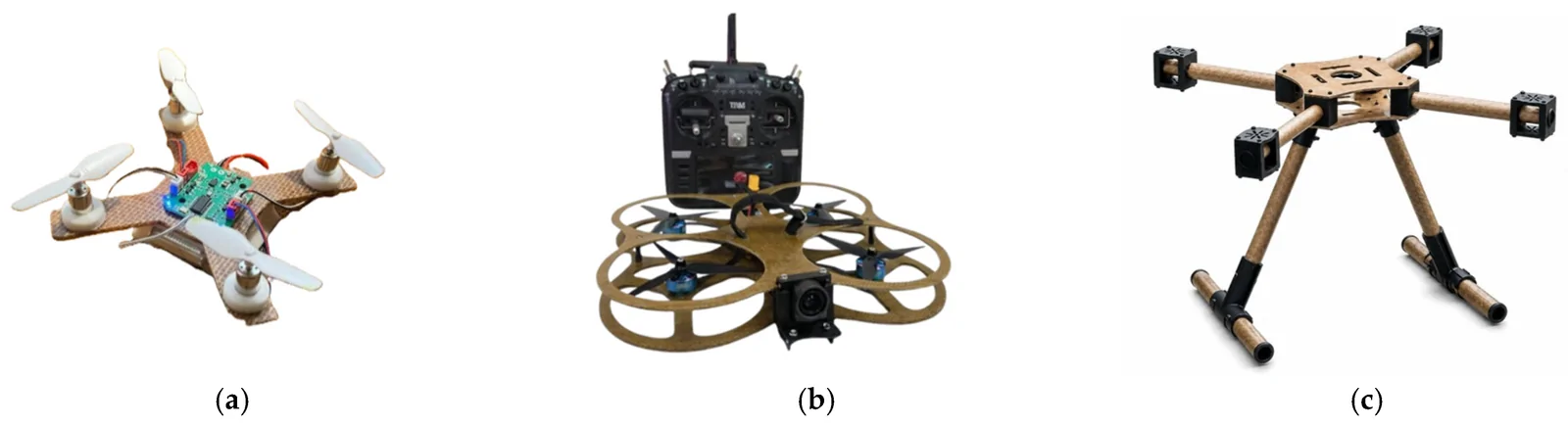
KFRC quadcopter platforms classified by physical scale and frame design: (**a**) micro-scale quadcopter utilising brushed motors for indoor/confined flights; (**b**) mid-scale ducted Cinewhoop quadcopter designed for safe close-proximity operations; (**c**) large-scale quadcopter featuring tubular structural arms and landing gear for high-payload capacity.

KNFRP, while weaker than synthetic FRPs, is lightweight, biodegradable and eco-friendly, making it a promising alternative for low-cost, disposable UAVs, surveillance drones or agricultural UAVs, where sustainability and reduced environmental impact are priorities. It performs well in low-to-moderate load conditions and can replace synthetic composites in non-critical structural parts like panels, fairings and internal reinforcements. The advantage of KNFRP lies in its renewable nature, lower carbon footprint and cost-effectiveness, making it a viable alternative for green aviation and low-impact UAV applications while maintaining a balance between performance and sustainability. Figure 2 shows ongoing efforts by Malaysian companies, such as Suxus SDN BHD [42], that have demonstrated the viability of KFRCs through prototypes such as quadcopter frames and other structural components.

Another Malaysian company, Midwest Composites SDN BHD, has successfully developed and fabricated hybrid kenaf and glass fibre-based UAV fuselages, wings, flaps and agricultural drone components, further demonstrating the commercial and structural feasibility of hybrid KFRCs for UAV applications [51]. For medium to high-load structural components, hybrid composites such as glass-kenaf hybrids or other natural fibre hybrids offer a practical solution [52]. These hybrid composites leverage the high strength and stiffness of synthetic fibres like glass, combined with the sustainability and weight advantages of KFs. However, the incorporation of glass fibre introduces added weight, partially offsetting the weight-saving benefits of using natural fibres. Despite this trade-off, hybrid KFRC provides a balanced approach to achieving the mechanical performance required for critical UAV components, such as payload structures, landing gear supports and motor mounts, while maintaining a degree of sustainability. This versatility makes KFRC and its hybrids ideal for structural innovations in UAV design. Table 2 shows previous research on KFRC and hybrid KFRC.

Hayeemasae et al. [53] developed kenaf fibre-reinforced composites by blending kenaf with recycled high-density polyethylene and NR. At an optimal fibre loading of 10 phr, the composite exhibited a 25% increase in tensile strength retention and a 5% improvement in elongation at break retention after 12 months of aging. This long-term mechanical stability is particularly important for UAV applications where drones are exposed to outdoor conditions for extended periods. Such materials could be ideal for external UAV body panels, flexible covers or lightweight aerodynamic fairings, offering both durability and environmental resilience. Setyayunita et al. [54] investigated the effect of NaCl solution treatments on kenaf/epoxy composites. With 5 wt.% NaCl treatment, the composite achieved a flexural strength of 2.085 GPa, flexural modulus of 19.77 MPa, internal bonding strength of 1.8 MPa, thickness swelling of 3% and water absorption of 10.9%. The exceptionally high flexural strength and good dimensional stability suggest this material is highly suitable for UAV internal structures requiring high stiffness-to-weight ratios, such as load-bearing beams, spars or skeleton frames that maintain integrity under aerodynamic loads and moderate humidity. Azammi et al. [55] developed a kenaf-filled composite using NR and TPU. The optimal blend, containing 12.5% kenaf, 50% NR and 37.5% TPU, exhibited low water absorption (<1%), density of 1.03 g/cm^3^, thickness swelling of 4% and a damping factor (tan *δ*) greater than 0.3. The excellent damping behaviour and low water uptake make these composites highly suitable for UAV vibration-damping applications, such as mounting platforms for sensitive avionics, battery supports or impact-resistant landing gear components.

Moreover, the development of hybrid KFRCs, where KFs are combined with other natural fibres and synthetic fibres in matrices such as vinyl ester, polyester and epoxy resins has resulted in composites with superior tensile, flexural and impact properties. By optimising the fibre-to-matrix ratio and laminate configurations, these hybrids offer enhanced strength-to-weight ratios and durability, meeting the stringent mechanical requirements for UAV parts such as load-bearing airframes, structural skins and modular payload compartments. Jothiprakash et al. [56] explored hybrid composites made of PALF, KF and VE matrices. The best combination, 20PALF/20KF/60VE, resulted in strong mechanical properties: tensile strength of 44 MPa, flexural strength of 43 MPa, compressive strength of 114 MPa, impact strength of 58.9 kJ/m^2^, microhardness of 39.5 HV and a specific wear rate of 9.7 × 10^5^ mm^3^/nm with a coefficient of friction of 0.213. These properties suggest that this hybrid system is well-suited for UAV structures requiring high impact resistance and wear durability, such as drone rotor guards, crash-resistant undercarriages or hybrid composite shells where mechanical toughness and abrasion resistance are vital. Afolabi et al. [57] evaluated the mechanical performance of sandwich composites with a syntactic foam core and hybrid kenaf/glass fibre face-sheets arranged in different stacking sequences. The study found that hybrid configurations significantly enhanced mechanical properties, with the glass-kenaf face-sheet achieving the highest tensile (40.39 MPa) and flexural (159.51 MPa) strengths, while the kenaf-glass face-sheet showed the highest compressive strength (63.81 MPa).

Azlin et al. [58] fabricated hybrid kenaf/polyester fibre/PLA laminates with varying layups. The best laminate (S5: K/K/P/K/K configuration) showed a *T_g_* of 58.90 °C, *T_m_* of 95.49 °C and a burning rate of 29 mm/min. The elevated *T_g_* and controlled thermal behaviour are advantageous for UAV applications where operating temperatures can fluctuate, making these laminates suitable for mid-temperature structural components such as UAV fuselage skins, battery compartments, and payload covers. Loganathan et al. [59] produced a hybrid composite by combining *Cyrtostachys renda* (red palm) fibre, KF, multi-walled carbon nanotubes and phenolic resin. The best formulation (5C:5K) delivered impressive mechanical results: tensile strength of 47.96 MPa, flexural strength of 84.61 MPa, impact strength of 7.28 kJ/m^2^ and water absorption of 26.93%. Although water absorption was relatively high, the enhanced mechanical strength makes this composite promising for semi-structural UAV parts such as rotor arms, engine mounting brackets or protective casings, if water exposure is managed through sealing or coating strategies. These advancements align closely with the evolving demand for eco-friendly, high-performance materials in next-generation UAVs, where reducing overall mass while maintaining strength, reliability and sustainability is paramount. Collectively, the research findings reinforce the viability of KFRC and hybrid KFRC systems as sustainable alternatives to traditional synthetic composites for complex UAV component manufacturing.

### 2.2. KNFRP Pellets for Complex Shape UAV Components

**Figure 3:**
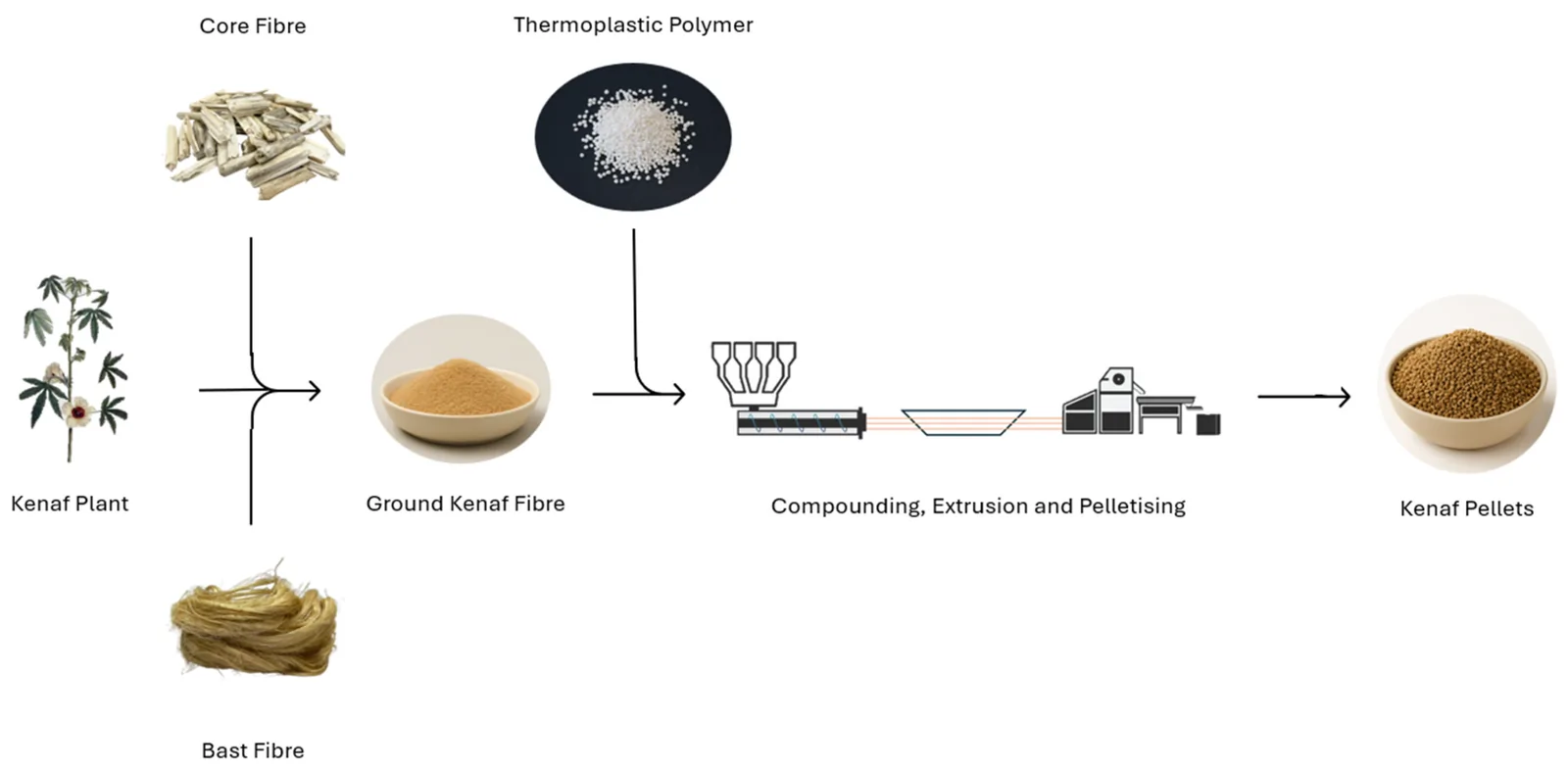
Kenaf pellets fabrication workflow.

KNFRP pellets are emerging as a sustainable solution for manufacturing UAV components with complex geometries, offering a balance between lightweight properties and structural integrity. These BC pellets are made by reinforcing thermoplastic matrices with KFs to create durable, lightweight and eco-friendly alternatives to traditional wooden or plastic pellets (Figure 3). These pellets utilise kenaf in different forms, either as kenaf powder, kenaf bast fibres or kenaf core particles, to enhance strength and impact resistance while maintaining recyclability. The processing of KNFRP typically involves compounding and moulding techniques such as injection moulding, extrusion or compression moulding, where KFs or powder are mixed with molten plastic and then shaped into pellets. The thermoplastic matrices commonly used include PP, polyethylene (PE), PLA and polyvinyl chloride, which offer good toughness, moisture resistance and recyclability. Kenaf processing depends on its form, where kenaf powder is obtained by grinding and sieving fibres into fine particles, kenaf bast fibres are mechanically separated and treated for better adhesion to plastics, and kenaf core, the inner woody part, is often chipped and refined for lightweight composite applications.

Injection moulding is ideal for producing precise and intricate components like casings and structural supports, while extrusion moulding is suited for continuous profiles like rods and beams. Compression moulding excels in creating large, flat or thick parts, such as panels, with excellent strength and low material waste. Advanced techniques like vacuum-assisted resin transfer moulding provide superior resin distribution and surface quality, making it suitable for high-performance aerospace components. Additionally, blow moulding and rotational moulding (roto-moulding) enable the production of lightweight, hollow parts like fuel tanks or enclosures. Each technique highlights the versatility of KNFRP in addressing the diverse requirements of UAV components while promoting sustainable manufacturing practices. Recent advancements in KNFRP pellets have revealed significant improvements in mechanical, thermal and flame-retardant properties through optimised material combinations and processing techniques, as listed in Table 3.

Zaki et al. [60] investigated the effects of adding coupling agents to K/PP pellets. The incorporation of MAPP into the K/PP composite increased tensile strength from 22.4 MPa to 29.3 MPa, reduced water absorption from 1.31% to 1.05% and improved impact resistance by enhancing fibre–matrix adhesion. SEM analysis confirmed better interfacial bonding, while the lower water uptake indicates improved durability under humid conditions. These results suggest that K/MAPP/PP composites are promising candidates for fabricating lightweight, impact-resistant UAV components such as fuselage panels, interior supports and protective housing where mechanical strength and moisture resistance are essential. Khan et al. [61] developed bamboo fibre- and KF-reinforced PLA hybrid composites, where BF-PLA composites achieved the highest tensile strength (25.95 MPa) and compressive strength (173.15 MPa), BF30-KF70 composites exhibited the best impact strength (6.6 Nm) and highest thermal degradation temperature (484 °C), and KF-PLA composites showed superior hardness resistance (69.2 HRS). The high tensile and compressive strengths of BF-PLA composites make them suitable for UAV load-bearing frames and mounting structures, while the excellent impact resistance and thermal stability of BF30-KF70 composites are ideal for UAV protective housings and crash-resistant components.

Tamta and Palsule [62] developed KF-reinforced chemically functionalised styrene acrylonitrile composites via extrusion and injection moulding. By varying the fibre loading, they found that the optimum formulation at 30/70 KF/CF-SAN resulted in remarkable mechanical enhancements such as an increase of 78% in tensile strength, a 75% increase in tensile modulus, a 125% increase in flexural strength and a 104% increase in flexural modulus compared to the neat matrix. These substantial gains indicate the potential of kenaf composites for use in high-stress UAV structural components, where the strength-to-weight ratio is crucial. Hassan et al. [63] developed a PLA/rHDPE/K composite foamed with ADC, achieving a flexural strength of 6.61 MPa, a flexural modulus of 3052.22 MPa, thermal stability with 10% weight loss at 300.9 °C and 9.4% burnt residue, making it a promising lightweight and thermally stable material for non-structural UAV components such as internal panels, insulation layers, and aerodynamic fairings where reduced weight and moderate mechanical strength are essential. Similarly, Woo and Cho [64] studied the effects of incorporating APP as a flame retardant in chopped K/PLA composites using extrusion and compression moulding. Their results demonstrated that with 50 parts of APP loading, the composites achieved a limiting oxygen index of 89%, a 32% increase in tensile modulus and an outstanding 165% improvement in storage modulus. These findings underline the suitability of K/PLA/APP composites for UAV parts requiring both mechanical integrity and enhanced fire safety, such as cargo compartments and battery enclosures.

Mustapa et al. [65] explored kenaf core fibre-reinforced PP composites fabricated via extrusion and compression moulding. They observed that increasing fibre loading improved flexural properties up to 20 wt.%, with a peak flexural strength of 48.37 MPa and a flexural modulus of 1.76 GPa. These enhancements suggest that kenaf core fibre/PP composites could serve well in semi-structural UAV parts where moderate loading and stiffness are necessary. Azam et al. [66] investigated a hybrid system by combining multiwalled carbon nanotubes with short KF-reinforced PP composites via injection moulding. By optimising fibre and nanotube loading (30 wt.% KF and 3 wt.% MWCNTs), the hybrid composite exhibited a water absorption rate of 8%, indicating improved water resistance while maintaining a relatively hydrophilic character beneficial for environmental exposure management. Furthermore, the flame retardancy was notably improved, with a flammability rate of 11 mm/min, suggesting the material could be advantageous for fire-sensitive UAV components. Another study was conducted by Hassan et al. [67] on foam composites fabricated via extrusion followed by hot and cold press moulding. The researchers developed foamed PLA/KF BCs using ADC as a blowing agent and investigated the effects of extrusion temperatures and ADC loadings on their performance. The study found that the lower-temperature profile (P2) and 3 phr ADC-incorporated kenaf foams showed improved mechanical strength (~5 MPa), lower moisture content (2.8%) and an onset temperature of 265 °C, making them suitable for lightweight, thermally stable UAV insulation and casing applications. Overall, these studies demonstrate that kenaf-based pellets, especially when optimised with functional additives like MWCNTs or APP and processed with appropriate fibre loadings, can achieve outstanding mechanical, thermal and fire-resistant properties. Such performance enhancements align with the critical needs of the UAV and drone industries, offering opportunities for sustainable, lightweight and high-performance material solutions.

### 2.3. KNFRP Filaments for Additive Manufacturing

Emerging technologies such as 3D printing using kenaf-based filaments [68] are also being explored for their ability to create complex and customised designs efficiently. Additive manufacturing (AM) with kenaf composites offers a sustainable approach to fabricating complex UAV components, enabling on-demand production and design flexibility. The process supports the creation of intricate geometries, lightweight structures and customised designs that are otherwise challenging with traditional manufacturing. According to Kantaros et al. [69], the use of composite filaments such as carbon fibre-reinforced PLA, polyethylene terephthalate glycol and nylon in 3D printing has significantly improved UAV manufacturing by enabling lightweight components with high strength, better structural performance and greater design flexibility. Building on this, KF has strong potential as a sustainable natural reinforcement in 3D printing filaments for UAV applications, where its low density, good specific mechanical properties, biodegradability and renewability can contribute to lighter, eco-friendly drone components while reducing dependence on synthetic fibres. The layer-by-layer fabrication minimises material waste, enhancing cost-efficiency and sustainability. Research to date on AM of kenaf composites is summarised in Table 4.

Alaa et al. [70] investigated 10 wt.% kenaf/PLA composites with untreated and superheated steam-treated KFs. The untreated kenaf composites achieved a tensile strength of 27.27 MPa, while the superheated steam-treated kenaf showed significant enhancement in dynamic mechanical properties, with a dynamic mechanical analysis modulus of 222.15 MPa and reduced rate of water absorption at 14.81%, indicating better stability in humid environments, critical for UAV parts operating in outdoor conditions. Hamat et al. [71] demonstrated that using a self-polymerised polydopamine coating on PLA/Kenaf composites improved mechanical properties remarkably. At 5 wt.% fibre loading, they achieved a tensile strength of 54.6 MPa and a flexural strength of 75.2 MPa, while higher loading (20 wt.%) resulted in a compressive strength of 108.6 MPa. This suggests PDA-treated composites are ideal for UAV components that require both flexibility (such as fairings) and impact resistance (such as landing gear skids). Lau et al. [72] evaluated kenaf/PLA composites at various fibre loadings, with the best mechanical performance at 15 wt.% fibre content, reaching a tensile strength of 47.02 MPa, a tensile modulus of 65.95 GPa and a maximum strain of 3.71%, offering a good balance of stiffness and ductility. These properties make them suitable for lightweight UAV body panels and aerodynamic surfaces.

Shahar et al. [73] highlighted that 3 wt.% kenaf/PLA composites exhibited the highest tensile strength of 54.78 MPa and an impact resistance of 3.19 kJ/m^2^, with the 7 wt.% composites showing superior fatigue life. This behaviour is crucial for UAVs subjected to repeated dynamic stresses, making these materials excellent candidates for rotor shrouds and vibration-damping parts. Khalid et al. [74] optimised kenaf/PP composites by applying 6% NaOH fibre treatment, achieving outstanding tensile strength of 267.69 MPa, tensile modulus of 11.88 GPa and shear modulus of 769.3 MPa at an injection temperature of 200 °C. The extremely high tensile strength and good fracture strain (2.07%) position these composites for load-bearing UAV components such as arm structures, fuselage skeletons and motor mounts. Finally, Aumnate et al. [75] developed PLA/Kenaf cellulose composites modified with tetraethyl orthosilicate and polyethylene glycol, achieving a tensile strength of around 45 MPa, a *T_g_* of 57.6 °C and a water contact angle of 81.1°, indicating improved hydrophobicity. These composites are particularly well-suited for 3D-printed UAV casings, lightweight enclosures and modular payload compartments where thermal stability and water resistance are important.

**Figure 4:**
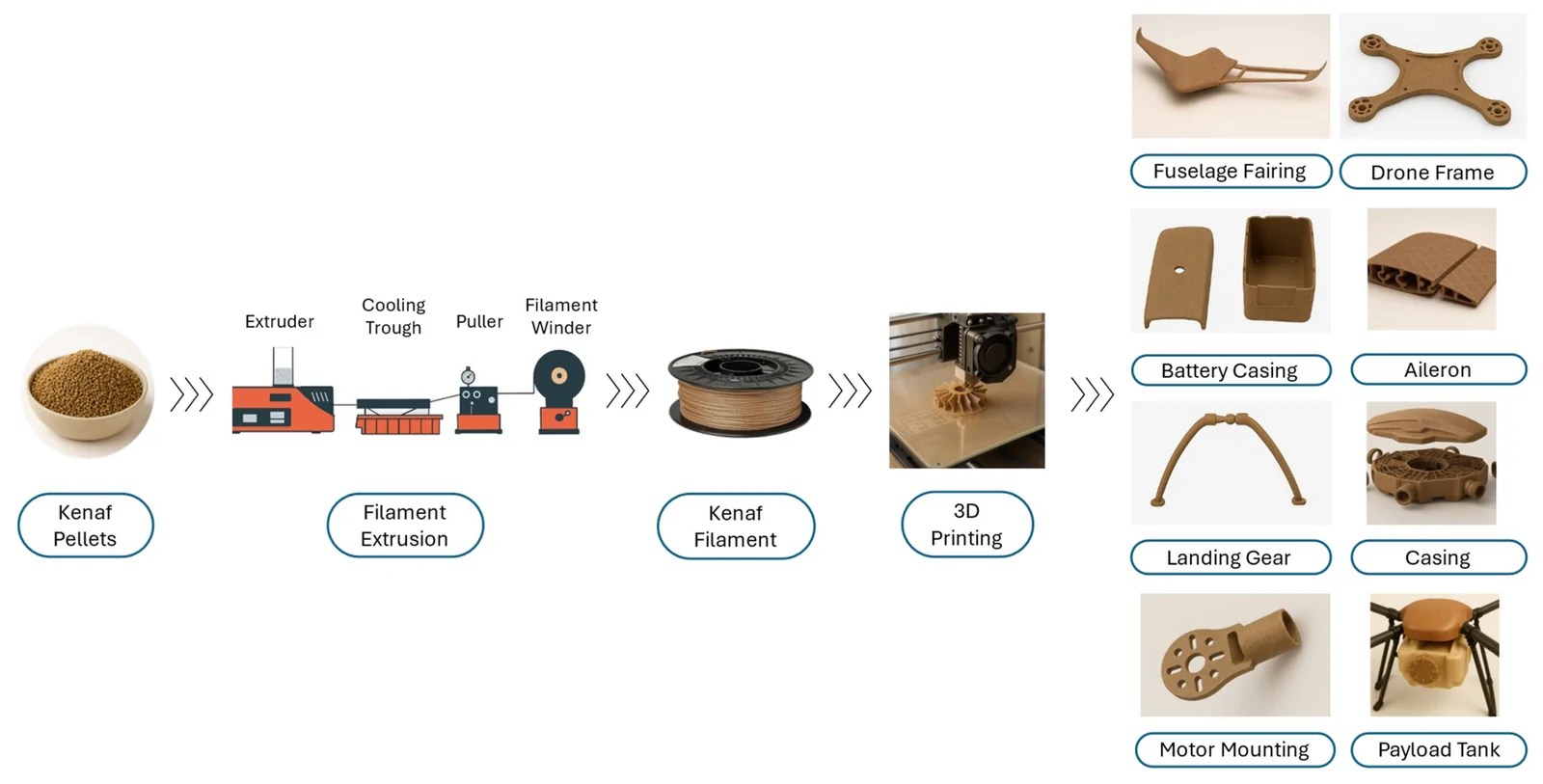
3D printing kenaf filament workflow.

Figure 4 illustrates the process of converting kenaf pellets into 3D-printed drone components through filament extrusion and AM. It shows key stages including extrusion, filament winding, and 3D printing, culminating in parts such as the fuselage fairing, drone frame, and payload tank. However, several challenges arise when using kenaf composites in AM for UAV components. The high thermal degradation risk of KFs during printing limits the choice of compatible thermoplastic or thermosetting matrices. Uneven fibre distribution in the composite feedstock can lead to inconsistent mechanical properties in the final print. Moisture absorption by KFs during storage or printing may lead to interlayer adhesion issues and reduced dimensional stability. Additionally, achieving high-performance properties, such as thermal stability and impact resistance, required for UAV applications is a challenge. Processing parameters in AM, such as extrusion temperature and nozzle clogging due to fibre agglomeration, also pose operational difficulties.

Processing parameters in AM, as well as during filament compounding and extrusion, play a crucial role in determining the printability and final performance of KF-reinforced thermoplastic composites. Based on the literature, pellet compounding using twin-screw extruders is commonly conducted at processing temperatures ranging from 170 to 190 °C with screw speeds of approximately 15 rpm. Alternative compounding methods have also been reported, including the use of a micro-compounder at 180 °C and 40 rpm, and a blade rotor mixer operating at 190 °C, 45 rpm and 25 min mixing time for homogenising KFs with polymer matrices. For filament fabrication using a single-screw extruder, processing temperatures typically range between 170 and 190 °C with screw speeds of 5–7 rpm. During fused deposition modelling (FDM) printing, nozzle temperatures are generally maintained between 200 and 210 °C, while bed temperatures range from 50 to 60 °C to ensure adequate layer adhesion and dimensional stability.

Processing parameters in AM, such as extrusion temperature and nozzle clogging due to fibre agglomeration, also pose operational difficulties. Printing temperature must be carefully controlled to ensure adequate polymer flow while avoiding thermal degradation of the lignocellulosic fibres [76]. Excessive temperatures can promote degradation of hemicellulose and cellulose constituents, leading to reduced fibre integrity and diminished mechanical performance [77]. Nozzle diameter is another critical parameter, as smaller nozzles may increase the likelihood of clogging when fibre length, particle size or fibre agglomeration exceeds the nozzle opening [78]. Current studies have employed nozzle diameters ranging from 0.4 to 0.8 mm, with larger nozzles generally providing more reliable extrusion of fibre-filled filaments. Furthermore, the diameter of bast KFs used in AM applications has been reported to range between 50 and 270 μm, with finer fibres being preferred because they disperse more uniformly within the polymer matrix, improve extrusion stability and reduce the risk of nozzle blockage during printing.

To overcome these challenges, surface-treated KFs (e.g., silane or alkali-treated) can be used to enhance their compatibility with the matrix and reduce moisture sensitivity. Low-temperature polymers such as PLA or specially formulated bio-resins can be employed to prevent fibre degradation during printing. Advanced feedstock preparation techniques, such as pre-compounding fibres with polymer or using uniformly dispersed filament extrusion, can improve fibre distribution and print quality. Introducing nanomaterials, such as carbon nanotubes or graphene, into the kenaf composite matrix can enhance thermal and mechanical performance. Optimising AM parameters, such as extrusion speed, layer thickness, and cooling rate, can help reduce defects and improve interlayer bonding. Protective post-processing treatments, such as coatings or annealing, can further enhance the durability and stability of the printed UAV components.

### 2.4. Kenaf-Based Paper, Boards, Foam and Biofilms for Insulation, Sound and Vibration Damping in UAV Electronics

Kenaf Pressed Paper (KPP), kenaf composite board (KCB), kenaf foam (KFM) and kenaf biofilm (KBF) offer significant sustainability benefits for use in UAVs, as they are made from renewable, biodegradable materials. KFs provide inherent electrical insulation and are eco-friendly, aligning with increasing industry emphasis on green technology and circular economy principles. As listed in Table 5, recent studies have highlighted the promising role of kenaf-based materials for insulation, sound, and vibration damping in UAV electronics. The applications include PCBs, sensor supports and thermal insulation layers.

Hashim et al. [79] developed KPP and compared it against conventional KPP. Although the tensile strength of KPP was slightly lower (48 MPa, about 8% reduction), it exhibited superior thermal stability (345 °C, 2.87% higher) and maintained a low relative permittivity (2.41 at 50 Hz), making it suitable for UAV electronic insulators and lightweight PCB substrates where thermal resistance and dielectric performance are critical. Umair et al. [80] reinforced KPP with polyvinyl alcohol, finding that with 6% PVA concentration, the composites achieved a tensile index of 81.36 Nm/g, a burst index of 6.73 kPam^2^/g and a tear index of 5.89 mNm^2^/g. Importantly, the material demonstrated strong electrical insulation properties with an AC breakdown voltage of 36.30 kV and an AC breakdown strength of 157.76 kV/mm, emphasising its potential for high-voltage UAV electronics and PCB dielectric layers. Adnan et al. [81] explored a mixture of oil palm EFB fibres and kenaf in pressed paper composites. A 100% kenaf composition yielded the highest tensile strength at 65.88 MPa, a lightning impulse breakdown voltage of 31.47 kV/mm and an AC breakdown voltage of 47.77 kV, reinforcing the suitability of pure KPP for critical electronic insulation in UAV flight controllers and sensor boards.

In composite boards, Asri et al. [82] worked on kenaf particleboards bonded with phenol-formaldehyde (PF) resin and enhanced with 2% NaOH-treated fibres. The optimised composite, containing 10% resin, achieved a flexural strength of 3.35 MPa, a flexural modulus of 955.82 MPa and an internal bonding strength of 0.16 MPa. However, it showed relatively high thickness swelling (57.28%) and water absorption (144.21%). Although mechanical properties were moderate, the high water absorption limits direct structural use. Nevertheless, such boards could still serve well as internal non-structural panels or removable insulation covers within UAV fuselages where exposure to moisture is controlled. Zawawi et al. [83] found that Kenaf/PLA composites had a permittivity (ε’) of 3.3 down to 2.5 and a loss tangent below 0.1, suggesting excellent signal transmission properties, crucial for UAV wireless communication boards and antenna substrates.

Kenaf-based foam is a lightweight, porous composite material derived from KFs embedded in polymer or geopolymer matrices, offering a unique combination of biodegradability, mechanical resilience and thermal and acoustic insulation properties. Foam materials are particularly attractive in engineering applications due to their low density, energy absorption capacity and multifunctionality, making them ideal for use in UAV systems where weight savings, vibration damping and environmental sustainability are critical. Chong et al. [84] developed LFC reinforced with 0.6% TKF, reporting notable mechanical enhancements including a 4% increase in compressive strength, 56% in flexural strength and 62% in tensile strength. These improvements make LFC-TKF composites suitable for non-structural UAV applications, particularly in internal panelling and protective housings for electronic systems where moderate strength and weight-saving are critical. Afolabi et al. [85] examined core SFCs reinforced with various face-sheet configurations of kenaf and glass fibres. The hybrid GK configuration exhibited the highest hardness (49.8 Hv), minimal water absorption (2.118%) and good buoyancy properties, while the KG layout achieved the lowest sound pressure levels (128.58 dB). These results suggest strong potential for acoustic shielding and vibration isolation panels in UAV fuselages or near propeller mounts where noise and vibration management are crucial.

Samaei et al. [86] incorporated KFs into PU foams and optimised both loading and fibre length. At 1.2 wt.% and 8 mm fibre length, the composite achieved a sound absorption average of 0.65, representing a 35.41% improvement over neat PU. This makes kenaf/PU foam an excellent candidate for acoustic damping applications in UAVs, such as lining drone hoods or internal compartments to suppress motor noise and enhance stealth performance. Zhang et al. [87] fabricated GFF composites by adding TKF and black liquor waste, yielding thermal conductivity in the range of 0.118–0.248 W/m·K and compressive strengths between 1.6–19.9 MPa. This multi-functional foam can serve as thermal insulation and mechanical support for UAV electronic bays, helping to manage heat from power electronics while maintaining structural integrity.

Siagian et al. [88] incorporated CNF from kenaf into PU foams, with 3 wt.% loading yielding optimal performance. The compressive strength increased to 284.43 kPa, modulus to 7.32 MPa and flexural strength to 734.15 kPa. These results support the use of CNF/PU foam in vibration-isolating mounts and shock-absorbing enclosures for fragile avionics or sensors in UAV systems. Soberi et al. [89] formulated kenaf core-based polyurethane foam with EG and ATH, where the best formulation (PU/KF/6ATH/10EG) achieved a compressive strength around 0.2 MPa and modulus near 5 MPa. The inclusion of fire retardants and moderate mechanical strength suggests this foam is ideal for fire-resistant compartment linings or energy-absorbing structures in UAVs operating in harsh environments. Majid et al. [90] studied palm oil-based rigid polyurethane foam reinforced with varying KF loadings, finding 7.5 pphp to be optimal with a compressive strength of 0.8963 MPa. This eco-friendly and mechanically competent foam can be used in eco-conscious drone components, particularly for mounting platforms or equipment trays requiring rigidity.

In the domain of biodegradable films, Hazrol et al. [91] developed kenaf/corn starch biofilms where 0.6% KF loading achieved a tensile strength of 17.74 MPa, a tensile modulus of 1324.74 MPa and an elongation at break of 48.79%. With low moisture content (33.67%) and water solubility (~5.99%), these films could serve as eco-friendly sensor adhesives or disposable UAV electronics casings, particularly in single-use or low-cost drone missions. Oyekanmi et al. [92] enhanced CNF composites derived from kenaf bast fibres, achieving a tensile strength of 37.5 MPa, tensile modulus around 300 MPa, elongation at break around 42% and a high water contact angle of 91.8°, confirming high hydrophobicity. These properties are highly desirable for creating waterproof UAV electronic housing, flexible circuit layers or moisture-resistant drone sensors.

**Figure 5:**
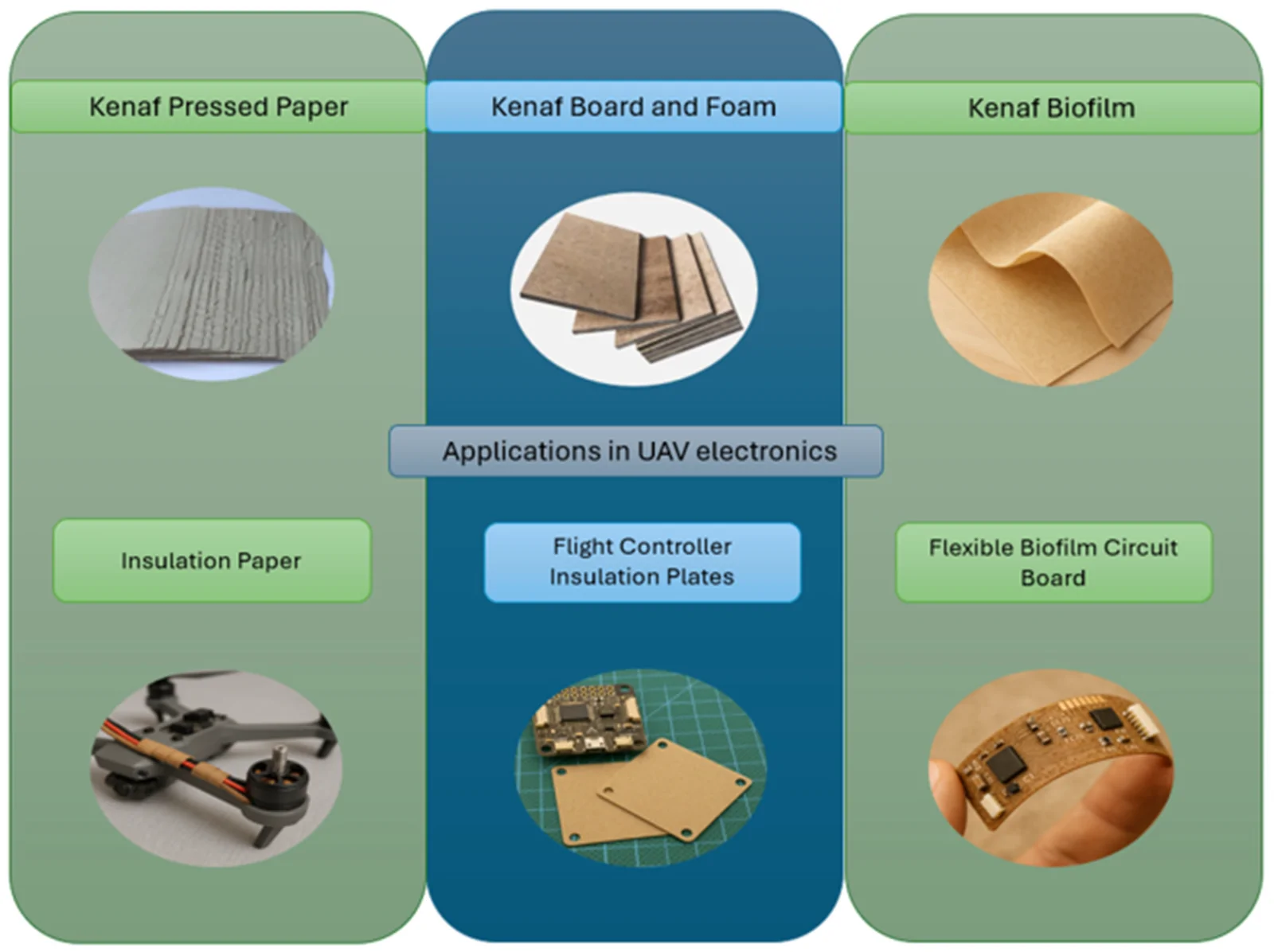
KPP, KCB, KFM and KBF for UAV electronics.

Figure 5 highlights the use of various kenaf-based materials such as pressed paper, board and foam, and biofilm for sustainable applications in UAV electronics. KPP is utilised to produce insulation paper, which serves as an effective barrier for electrical insulation and thermal protection in UAV motor components. Kenaf board and foam are transformed into rigid structures like flight controller insulation plates, offering mechanical support, electrical isolation and additional benefits such as sound and vibration damping, crucial for maintaining the stability and performance of UAV systems. Meanwhile, kenaf biofilm is engineered into flexible circuit boards, providing an eco-friendly alternative for lightweight and compact electronic integration. Collectively, these applications demonstrate the multifunctionality and environmental benefits of kenaf materials in advancing green aerospace technology, especially in electronic protection, acoustic management and vibration control.

### 2.5. Summary of Achievements and Advantages of Kenaf-Based Materials for UAV Applications

Compared with conventional UAV materials, KNFRPs generally exhibit lower mechanical performance than CFRPs, GFRPs and aluminium alloys. CFRPs typically possess tensile strengths of 600–1500 MPa, elastic moduli of 70–150 GPa and densities of approximately 1.5–1.6 g/cm^3^, making them the preferred material for primary load-bearing UAV structures. GFRPs generally exhibit tensile strengths ranging from 200 to 900 MPa with elastic moduli of 20–50 GPa and densities of 1.8–2.0 g/cm^3^, providing an effective balance between strength and manufacturing cost. Aluminium 6061-T6, commonly used for UAV frames and landing gear, has a typical ultimate tensile strength of approximately 310 MPa, an elastic modulus of approximately 69 GPa and a density of 2.7 g/cm^3^.

In comparison, the studies reviewed in this work reported tensile strengths of 40–270 MPa, flexural strengths exceeding 150 MPa, compressive strengths approaching 170 MPa, and densities typically ranging from 1.2 to 1.4 g/cm^3^ for kenaf-based composites. Although these values are generally lower than those of conventional aerospace composites, kenaf composites offer significant advantages in terms of reduced weight, renewable sourcing, biodegradability, vibration damping and a substantially lower environmental footprint.

Despite their comparatively lower mechanical properties, the reported performance demonstrates that KNFRPs can satisfy the requirements of numerous non-critical and semi-structural UAV components. Their tensile strengths of up to 270 MPa and flexural strengths exceeding 150 MPa are adequate for components subjected to moderate loading, including fuselage shells, fairings, payload covers, battery enclosures, electronic housings and internal support structures. Furthermore, hybrid kenaf composites reinforced with glass fibres have achieved flexural strengths of approximately 160 MPa and impact strengths approaching 59 kJ/m^2^, extending their applicability to moderately loaded components such as UAV arms, landing gear supports and motor mounts. These findings indicate that kenaf-based composites should not be considered direct replacements for CFRPs in highly loaded primary structures; instead, they represent sustainable engineering materials whose deployment should be matched to the mechanical demands and functional requirements of specific UAV components.

For UAV arms and booms, the primary loading conditions are bending and torsion generated by propeller thrust, manoeuvring forces and landing loads. The reported tensile and flexural strengths suggest that kenaf-based composites possess sufficient stiffness and strength for small- to medium-sized UAV arms, although further validation through static, modal and fatigue analyses is required. Fuselage shells are typically subjected to distributed bending loads, vibration and local impact during operation. The favourable strength-to-weight ratio and vibration-damping characteristics of kenaf composites indicate their suitability for lightweight fuselage structures. Internal support structures, brackets and electronic housings generally experience lower structural demands and are therefore among the most promising near-term applications for kenaf-based materials.

For electronic and electrical applications, kenaf-based insulating materials have demonstrated dielectric constants of approximately 2.5, electrical breakdown strengths exceeding 150 kV/mm and thermal stability approaching 350 °C. These properties compare favourably with conventional insulating materials used in electronic enclosures and insulation systems, supporting their use in UAV electronic housings, battery compartment liners and electrical insulation components.

The successful implementation of kenaf-based materials in UAV systems requires component-specific evaluation through tensile, flexural, compressive, impact, vibration, fatigue and torsional testing, supported by FEA to assess structural integrity under realistic operating conditions. Collectively, the reported achievements demonstrate the potential of kenaf-based materials to fulfil both structural and electrical functional requirements while offering significant sustainability benefits for next-generation UAVs.

## 3. Challenges and Mitigation Strategies

The application of KF in UAVs presents both opportunities and challenges in terms of performance, sustainability and scalability. While KF offers eco-friendly advantages, its adoption is hindered by limitations in performance, as well as challenges related to supply chain demands and recyclability. Addressing these issues through material enhancements, advanced processing techniques and strategic industry initiatives is essential for integrating kenaf composites into UAV applications successfully.

### 3.1. Long-Term Environmental Durability of KNFRP for UAV Applications

KNFRP in UAV structures are exposed to multiple environmental stressors during service, including moisture, ultraviolet (UV) radiation, atmospheric oxidation, temperature fluctuations and biological degradation. These factors can progressively alter the physical, mechanical and electrical properties of the composite. As UAVs are frequently operated in outdoor environments and are often expected to maintain reliable performance over extended periods, understanding the environmental factors that influence KNFRP is essential for assessing structural integrity, operational safety and service life.

#### 3.1.1. Moisture Absorption

One of the primary drawbacks of KFs is their hydrophilic nature, meaning they absorb moisture from the environment. KFs contain high amounts of cellulose and hemicellulose, which possess numerous hydroxyl (-OH) groups capable of attracting and retaining moisture [93]. Consequently, kenaf-based composites, boards, papers and foams are susceptible to water absorption when exposed to humid environments, rainfall and cyclic wetting–drying conditions commonly encountered during UAV operation. Moisture absorption can induce fibre swelling and dimensional instability, generating internal stresses at the fibre–matrix interface and weakening interfacial adhesion. The resulting microcracks and interfacial debonding reduce the efficiency of load transfer between the KFs and surrounding matrix, leading to deterioration in tensile, flexural and impact performance [94]. For UAV structural components such as arms, fuselage shells and support structures, these effects may reduce stiffness, strength and vibration resistance, potentially compromising structural reliability during prolonged outdoor service [95].

The issue is also relevant for kenaf-based electrical and insulation materials. Moisture ingress can increase dielectric losses, reduce electrical insulation performance and accelerate ageing of paper-based insulating systems. This concern is particularly important for UAV electronic housings and insulation components operating in tropical climates characterised by high humidity and frequent rainfall. According to Fukuhara et al. [96], the cellulose-rich composition of KFs can absorb moisture up to approximately 17.5 wt.% of their dry weight, significantly influencing their dielectric and electrical conduction behaviour. The study showed that absorbed water forms quasi-solid layers on cellulose nanofibrils, increasing electrical conduction and potentially reducing the long-term insulation performance of kenaf-based electrical and electronic components under humid operating conditions. To mitigate moisture-induced deterioration of electrical insulation performance, Hussain and Sofiyan [97] incorporated polyvinyl alcohol into oil-impregnated kenaf paper and demonstrated improved dielectric breakdown strength, enhanced thermal stability and reduced moisture absorption compared with untreated kenaf paper. The findings suggest that polymer modification can effectively improve the resistance of kenaf-based insulating materials to moisture ingress, thereby enhancing their long-term dielectric performance and operational reliability.

Previous studies have reported the use of alkali treatment, silane treatment, acetylation, coupling agents and hydrophobic coatings [98] to reduce moisture uptake and enhance fibre–matrix compatibility. Other strategies to mitigate moisture absorption in kenaf composites include the incorporation of nanofillers. Ganvir and Ganvir [99] investigated the incorporation of 2 wt.% surface-modified bentonite nano-clay into kenaf/epoxy composites as a strategy to mitigate moisture absorption and improve environmental durability. The addition of nano-clay reduced the saturation moisture uptake by 52% and the diffusion coefficient by 73%, while effectively preserving the tensile and flexural properties of the composites after artificial seawater exposure, demonstrating its potential to enhance the long-term durability of kenaf-based UAV structures in humid environments. Future research should therefore prioritize the development of moisture-resistant kenaf-based materials while maintaining the lightweight, mechanical and insulating properties required for sustainable UAV applications.

#### 3.1.2. Ultraviolet, Oxidative and Thermal Ageing

During outdoor operation, UAV structures are continuously exposed to solar UV radiation, atmospheric oxygen and cyclic temperature variations. Prolonged UV exposure can initiate photo-oxidative degradation within the polymer matrix, resulting in chain scission, surface cracking, discoloration and reduced interfacial bonding between KFs and the matrix [100]. Similar degradation behaviour has been reported in kenaf-based composites subjected to accelerated weathering conditions. Azam et al. [101] investigated long unidirectional kenaf/PLA composites and reported that although kenaf reinforcement improved the weathering resistance of PLA, prolonged UV exposure still caused strength degradation due to matrix deterioration and structural imperfections. Likewise, Hassanuthin et al. [102] demonstrated that extended UV exposure reduced the tensile strength of kenaf/polypropylene composites reinforced with rice husk ash silica, confirming that UV radiation can adversely affect the mechanical integrity of kenaf composites. Simultaneously, thermal cycling caused by day–night temperature fluctuations can generate residual stresses due to differences in the thermal expansion behaviour of fibres and polymers. These degradation mechanisms may progressively reduce tensile, flexural and impact properties, leading to embrittlement and diminished load-bearing capability over extended service periods.

For UAV structural components such as arms, fuselage shells and support frames, environmental ageing may compromise structural stiffness, vibration resistance and fatigue performance. Applying UV-resistant and bio-based protective coatings can enhance durability and prevent environmental degradation. Riyadh et al. [103] employed nanocarbon-enhanced UV-curable polyurethane coatings containing graphene nanoplatelets and multi-walled carbon nanotubes to improve the environmental durability of recycled polyvinyl chloride/kenaf composites. At an optimal graphene nanoplatelets loading of 3 wt.%, the coating achieved a hydrophobic contact angle of 97° and retained over 97% of its flexural strength after accelerated weathering, demonstrating that nanocarbon-based protective coatings can effectively enhance moisture resistance, wear performance and long-term durability of kenaf-based composites. Additionally, post-curing and thermal stabilisation processes can further improve stiffness and long-term reliability, ensuring that kenaf composites maintain their structural integrity throughout the UAV’s operational life cycle. Future investigations should therefore incorporate accelerated weathering, UV ageing and thermal cycling tests to evaluate performance retention under realistic service conditions.

#### 3.1.3. Biodegradation and Microbial Attack in Humid Climates

While the biodegradability of KFs represents a significant environmental advantage, it may also present durability challenges during long-term storage and operation. The lignocellulosic nature of KFs makes them susceptible to biological degradation when exposed to moisture, high relative humidity and favourable microbial conditions. In tropical and subtropical climates, prolonged exposure to humid environments can promote the growth of mould, fungi and other microorganisms on fibre surfaces, particularly when moisture penetrates through matrix microcracks or interfacial defects. Joyyi et al. [104] investigated the biodegradation behaviour of KF-reinforced poly(3-hydroxybutyrate-co-3-hydroxyhexanoate) composites and highlighted the susceptibility of kenaf composites to microbial attack under soil conditions. The study reported that the incorporation of 30 wt.% KFs enhanced the mechanical performance of the biocomposites. However, the composites exhibited increased water absorption and accelerated weight loss during soil burial due to microbial degradation. Several microorganisms, including *Burkholderia* sp., *Streptomyces* sp., *Amycolatopsis* sp. and *Streptacidiphilus* sp. were identified as contributors to the biodegradation process, demonstrating that microbial activity can significantly influence the long-term durability and structural stability of kenaf-based composites in humid and biologically active environments. Similarly, Hidayat and Tachibana [105] investigated the biodegradation of PLA/kenaf composites using *Pleurotus ostreatus* and reported that fungal degradation progressively increased with exposure time, reaching 48% weight loss after 6 months. The degradation process caused significant deterioration of the composite structure, including fibre shortening and an 84% reduction in mechanical properties, attributed to enzymatic activity that enabled the breakdown of both KFs and the PLA matrix.

Biological degradation may accelerate fibre deterioration, weaken fibre–matrix adhesion and reduce the mechanical integrity of KNFRP structures. Furthermore, moisture-assisted microbial activity can contribute to dimensional instability, surface degradation and reductions in structural performance over time. These concerns are particularly relevant for UAV systems intended for agricultural monitoring, forestry surveillance and environmental monitoring, where operation frequently occurs under humid outdoor conditions. To overcome microbial attack in kenaf composites, several strategies can be implemented, including fibre treatments, incorporation of antimicrobial agents or nanoparticles, hydrophobic surface modification, matrix modification and protective coatings to reduce moisture uptake and inhibit microbial colonisation. Since there remains a significant research gap in understanding microbial degradation mechanisms and developing effective mitigation strategies for kenaf composites, future research should focus on systematically investigating microbial–composite interactions, identifying dominant degradation pathways and designing bio-resistant kenaf composite systems with improved long-term durability for outdoor and structural applications.

### 3.2. Mechanical Performance Limitations and Structural Applicability of KNFRP for UAV

Despite their favorable specific properties, low density and sustainability advantages, pure KNFRP composites generally exhibit lower absolute stiffness, tensile strength, compressive strength and impact resistance than conventional aerospace-grade carbon fibre composites. These limitations can restrict the ability of KNFRP structures to withstand high aerodynamic loads, cyclic fatigue, impact events and harsh operating conditions encountered during UAV missions. As a result, the direct substitution of carbon fibre composites with pure KNFRP in critical load-bearing structures remains challenging, particularly in applications requiring exceptional structural rigidity, dimensional stability and damage tolerance.

The suitability of pure KNFRP for UAV applications is therefore highly dependent on the operational requirements and loading conditions of the platform. Pure kenaf-based composites are generally more appropriate for low- to moderate-load, small-sized UAV systems [106], such as educational drones, recreational UAVs, environmental monitoring platforms, agricultural surveillance drones and short-range mapping aircraft, where lightweight construction, low manufacturing cost and environmental sustainability are prioritised. In these applications, KNFRP can be effectively utilised in secondary or semi-structural components, including fuselage shells, fairings, battery housings, payload compartments, landing gear covers and interior support structures. However, for larger UAVs, long-endurance platforms, heavy-payload delivery drones, high-speed aircraft and military-grade systems, the structural demands are substantially higher. Components such as wing spars, rotor blades, primary frames and load-bearing joints are subjected to significant static, dynamic and fatigue loads that may exceed the capabilities of pure kenaf composites, necessitating additional reinforcement strategies to ensure structural integrity and operational safety.

Hybridisation of KFs with high-performance reinforcements, such as carbon, glass, basalt or aramid fibres, has emerged as a promising strategy for enhancing stiffness, strength, impact resistance and fatigue durability while reducing the overall environmental footprint of the composite. Similarly, the incorporation of nanomaterials, including graphene, carbon nanotubes, nanocellulose and nanosilica can improve interfacial bonding and load transfer efficiency within the composite structure. Optimised laminate architectures, fibre orientation control and advanced manufacturing techniques can further enhance structural performance while minimising defects and weight penalties. For instance, the commercial viability of natural fibre composites has been demonstrated by Biscaro and Baldegger [107], who developed high-performance flax fibre reinforcement technologies (powerRibs™ and ampliTex™). These were successfully adopted in Formula 1 racing cars to replace selected carbon fibre components, achieving significant reductions in environmental impact while maintaining the stringent mechanical performance requirements of motorsport applications [108]. The successful adoption of flax-based composites in Formula 1 suggests that suitably engineered kenaf-based composites could also serve as sustainable alternatives for selected UAV components, particularly when combined with hybridisation and advanced manufacturing technologies to meet the required structural performance.

### 3.3. Thermal Stability of KNFRP for UAV Applications

KFs exhibit limited thermal stability compared with synthetic reinforcements due to their lignocellulosic composition, which consists mainly of cellulose, hemicellulose and lignin. In UAV applications, thermal exposure may occur due to aerodynamic heating, proximity to propulsion systems, battery-generated heat or high-speed flight conditions. Under prolonged thermal loading, KNFRP structures may experience fibre degradation, dimensional changes, reduced interfacial bonding and mechanical strength deterioration. Consequently, the application of KNFRP in high-temperature UAV components, such as engine housings, propulsion systems and heat-exposed structural regions, remains challenging unless appropriate thermal protection strategies are implemented. Thermal management strategies can support the integration of kenaf-based composites in UAV structures by mitigating heat exposure from battery systems, reducing the risk of thermally induced degradation and enabling the use of lightweight natural fibre composites in proximity to temperature-sensitive components. Hu et al. [109] proposed a hybrid battery thermal management strategy combining phase change materials with air cooling to improve the thermal safety of UAV lithium-ion batteries. The optimised system effectively reduced the maximum battery temperature by 43.3% while maintaining a lightweight design, demonstrating that integrating passive and active cooling mechanisms can provide an effective balance between thermal performance and weight constraints in UAV applications.

In addition to thermal degradation, dimensional instability caused by the mismatch in coefficients of thermal expansion between KFs and polymer matrices presents another critical concern. Differences in thermal expansion behaviour can induce internal stresses, microcracking, warping and shrinkage during manufacturing or service conditions. For UAV structures, even minor dimensional variations can negatively influence aerodynamic accuracy, component alignment, vibration behaviour and overall flight efficiency. Therefore, controlling thermal-induced deformation is essential for ensuring the reliability of kenaf-based composite components.

Several approaches have been explored to enhance the thermal stability of kenaf composites. Fibre surface modification and protective coatings have demonstrated potential in improving thermal resistance by enhancing fibre–matrix interactions and delaying degradation. Owen et al. [110] investigated the use of epoxy-coated KFs as a moisture-resistant reinforcement in recycled waste PET composites and reported enhanced interfacial bonding, mechanical performance and thermal stability. The epoxy coating increased the thermal degradation temperature from 397.0 °C to 409.4 °C and raised the melting peak to 252.8 °C, demonstrating that fibre surface coating is an effective strategy for improving the durability and environmental resistance of kenaf-based composites.

Beyond fibre modification, incorporation of thermally resistant hybrid reinforcements and insulating coatings offers additional opportunities for UAV applications. Chen et al. [111] developed a ceramic fibre–functionalised silica aerogel coating for UAV thermal protection, demonstrating an ultralow thermal conductivity of 0.032 W·m^−1^·K^−1^ and a compressive modulus of 2155.7 kPa, enabling effective thermal insulation while maintaining structural integrity at temperatures exceeding 1300 °C. The coating also exhibited excellent flame retardancy, UV stability and hydrophobicity, successfully protecting temperature-sensitive UAV components, particularly batteries, from high-temperature exposure without significantly increasing structural weight. Although such high-temperature coatings are beyond the requirements of conventional KNFRP structures, their design principles provide valuable insights for developing lightweight thermal barrier solutions for kenaf-based UAV components.

### 3.4. Manufacturing and Processing Challenges

Precision in composite manufacturing is crucial, as dimensional instability and warping issues can limit the application of kenaf composites in high-tolerance UAV components [112]. Producing high-performance UAV structures from kenaf composites requires precise fibre alignment, optimised resin infiltration and defect-free curing processes. Conventional manufacturing techniques, such as hand lay-up, often introduce manufacturing defects including void formation, air entrapment, uneven fibre distribution and incomplete fibre wetting, which can significantly reduce load transfer efficiency and compromise mechanical performance. Therefore, advanced manufacturing approaches, such as vacuum-assisted resin infusion, resin transfer moulding, compression moulding and automated fibre placement, are increasingly considered to improve composite quality and structural reliability. For instance, profilometry sensors and thermographic scanning techniques have been implemented to detect and correct manufacturing defects during automated fibre placement, improving laminate microstructure and reducing in-plane fibre waviness in the final composite structure [113].

However, the manufacturing of high-strength composites often requires elevated processing temperatures to achieve sufficient resin flow, consolidation and fibre–matrix adhesion. This presents a significant challenge for kenaf-based composites, as temperatures exceeding approximately 180–220 °C may initiate thermal degradation of the lignocellulosic fibres, causing cellulose decomposition, reduced fibre strength and deterioration of the fibre–matrix interface. Consequently, the processing window of kenaf composites is narrower compared with conventional synthetic fibre composites, complicating the fabrication of high-performance structures requiring high-temperature processing conditions. Processing temperatures are typically maintained below 200 °C to minimise fibre deterioration and preserve mechanical performance. Hence, the selection of suitable matrix materials, optimisation of curing parameters and development of low-temperature processing techniques are therefore critical for maintaining fibre integrity while achieving adequate composite consolidation.

Several strategies have been proposed to overcome these manufacturing limitations. The use of low-temperature-curing thermoset resins or thermoplastic matrices with lower processing temperatures, such as PP, PE and PLA can minimise thermal damage to KFs. Additionally, process optimisation through numerical simulation, real-time monitoring, and additive manufacturing-assisted fabrication could improve dimensional accuracy, minimise defects and enable more consistent production of kenaf-based UAV components. Nevertheless, balancing manufacturing complexity, processing cost and performance requirements remains a critical challenge for the large-scale adoption of kenaf composites in aerospace applications.

### 3.5. Recyclability of KNFRP

While kenaf-based composites are promoted as sustainable engineering materials, another key challenge is their recyclability when compared to metals like aluminium, which has a high resale value and well-established recycling infrastructure. Aluminium can be melted and reused indefinitely without significant degradation, making it highly attractive in aerospace applications. In contrast, KF composites face limitations due to the complexity of separating natural fibres from polymer matrices, which affects their recyclability and resale value. Thermoset-based kenaf composites, commonly used in UAV applications, are not easily recyclable as they do not remelt like thermoplastics, posing a challenge for sustainable disposal and reuse [114]. To address this issue, using thermoplastic matrices instead of thermosets can significantly improve the recyclability of kenaf-based composites. Thermoplastics such as PP, PLA and PE allow the composite to be shredded, melted and reformed into new components, creating a circular economy for UAV manufacturing. Additionally, by using biodegradable resins like PLA, UAV components made from kenaf can naturally break down under controlled composting conditions, reducing landfill waste and environmental impact. This approach ensures that even if recycling infrastructure is limited, kenaf-based materials still maintain an eco-friendly disposal advantage over synthetic composites.

Another promising solution is the development of hybrid recycling approaches that separate fibre from the polymer matrix. Chemical and mechanical recycling techniques, including solvent-based dissolution, pyrolysis and enzymatic treatments, can help break down the polymer while preserving KFs for reuse in secondary applications such as automotive interiors, packaging and non-structural UAV components. Ferjan et al. [115] mentioned that BCs offer environmental benefits for the aviation industry, but effective recycling remains a challenge, with solvolysis, pyrolysis and mechanical recycling emerging as the most promising technologies. Life cycle assessment (LCA) and techno-economic analysis highlight solvolysis as the most efficient option due to lower greenhouse gas emissions (17% less global warming potential) and cost advantages, emphasising the need for improved pre-treatment processes and optimised solvent management for greater circularity in aviation waste management. Investing in innovative fibre recovery methods can extend the lifecycle of kenaf-based materials, making them more competitive against metals like aluminium.

Lastly, establishing a structured recycling market for kenaf composites is essential. Unlike aluminium, which has a well-established resale value, kenaf-based materials need dedicated recycling initiatives. Governments and industries can introduce take-back programs, support recycling plants, and create demand for recycled kenaf composites in sectors such as automotive, construction and UAV manufacturing. By building a sustainable supply chain, kenaf composites can become a viable alternative to aluminium and synthetic fibre composites, ensuring their long-term adoption in aerospace and UAV industries.

### 3.6. Supply Meeting the Demand

One of the major challenges in applying KF in UAV manufacturing is the supply chain’s ability to meet the growing demand for high-quality, aviation-grade kenaf composites. As UAV technology advances and industries shift towards sustainable materials, the need for consistent, high-strength and well-processed KF increases. However, the availability of KF in sufficient quantities, uniform quality and aerospace-grade processing standards remains a concern [116]. Factors such as climate dependency, inconsistent fibre processing techniques, and limited large-scale industrial adoption hinder their widespread application in UAV composite manufacturing. A key solution to this issue lies in strategic kenaf cultivation and industrial processing, particularly in Malaysia, which has a thriving kenaf industry with government-backed initiatives and a favourable tropical climate for large-scale kenaf farming. Malaysia’s National Kenaf and Tobacco Board has been supporting kenaf-based industries and expanding cultivation, improving fibre extraction technologies, and establishing dedicated processing hubs, which can ensure a steady and reliable supply of kenaf for aerospace applications. A study by Alpandi et al. [117] on kenaf farming in Kelantan, Malaysia, used data envelopment analysis to assess energy efficiency, identifying the most and least efficient farmers. The findings suggest that kenaf farmers could save 11.3% of energy without reducing yield, with the highest energy-saving potential from fertilisers (86.5%), followed by diesel (13%), pesticides (0.3%) and human labour (0.2%).

Additionally, standardisation of KF treatment through chemical modification, hybridisation with synthetic fibres and better resin compatibility techniques can enhance its mechanical properties to meet UAV industry requirements. Investing in automation for fibre extraction and reinforcement, developing kenaf-based prepregs and integrating KF into existing composite supply chains will bridge the gap between raw material supply and high-performance UAV component demands. Nurhasanah et al. [118] studied the development of a blueprint for an Intelligent Decision Support System in the KF agroindustry supply chain in Indonesia, focusing on both upstream and middle-stream networks through simulation and digital platform development. This approach can serve as a valuable example for Malaysia to mitigate supply–demand challenges in its kenaf industry through better supply chain integration and decision-making support. With Malaysia’s strong market potential, ongoing research in BCs and sustainable aviation initiatives, the country is well-positioned to become a global leader in kenaf-based UAV materials, ensuring a consistent, scalable and high-performance supply chain for future aerospace applications.

## 4. Future Directions and Gaps

Despite promising developments in structural, insulation and AM applications, significant challenges remain regarding long-term durability, multifunctionality, manufacturing scalability, certification and industrial adoption. Addressing these gaps is essential for the successful deployment of KF in next-generation UAV systems.

### 4.1. Material and Manufacturing Innovations for Enhanced Performance

Nanotechnology presents a transformative opportunity to enhance the mechanical, thermal and electrical properties of kenaf-based composites for UAV applications. By integrating nanomaterials such as carbon nanotubes, graphene or silica nanoparticles into KF-reinforced polymers, significant improvements in strength-to-weight ratio, durability and conductivity can be achieved. According to a study by Malik et al. [119], incorporating 0.2 wt.% graphene nanoplatelets into KF-epoxy composites significantly enhanced mechanical properties, including tensile strength (30.5%), tensile modulus (61.5%) and interlaminar shear strength (35.1%), while also improving water absorption resistance by 7%. These enhancements make kenaf composites more competitive with conventional aerospace materials, enabling their use in critical UAV structures. However, most studies remain limited to coupon-scale mechanical testing, and the effectiveness of nanomaterial reinforcement under realistic UAV loading conditions, including vibration, fatigue and impact loading, remains largely unexplored.

Additionally, nanocoatings can provide moisture resistance and UV protection, mitigating one of the primary challenges associated with natural fibre composites. As research advances, the potential for nanotechnology-driven functionalisation, such as self-healing coatings and piezoelectric properties, could further revolutionise the application of kenaf in UAV design. Mohammed et al. [120] mentioned that surface treatment of KF with zinc oxide nanoparticles significantly improved its hydrophobicity and compatibility with a polymer matrix, with 2 wt.% zinc oxide nanoparticles yielding optimal results. The TKF composites exhibited higher contact angles, reduced water absorption and enhanced mechanical properties, as confirmed by analyses using scanning electron microscopy, Fourier transform infrared spectroscopy, X-ray diffraction and atomic force microscopy.

Another avenue for research is the development of high-performance bio-resins with improved thermal and mechanical stability. Biodegradable or partially bio-based epoxy [121,122] and thermoplastic matrices [123] could offer a balance between sustainability and high-performance characteristics. Exploring novel resin formulations that optimise adhesion between KFs and polymer matrices will be crucial for enhancing the overall mechanical integrity of UAV components. Rivadeneira-Velasco et al. [124] found that thermoplastic starch nanocomposites reinforced with nanofillers like natural montmorillonite, organically modified montmorillonite, cellulose nanocrystals and cellulose nanofibres exhibit enhanced mechanical strength, hydrophobicity and biodegradability, though with reduced transparency and increased colour variation. Proper dispersion and compatibility of nanofillers within the TPS matrix are crucial for optimising these properties, making TPS a promising eco-friendly bio-based matrix in kenaf composites.

Additionally, traditional composite manufacturing techniques, such as hand lay-up and vacuum-assisted resin transfer moulding, are widely used but often lead to inconsistencies in fibre distribution and void content. Future advancements should focus on automated and precision-based techniques such as AM and automated fibre placement [125]. 3D printing with kenaf-based filaments offers significant potential for producing complex, lightweight UAV components with minimal material waste. Hybrid processing approaches, such as combining resin infusion with 3D printing, could create multi-functional UAV components that integrate structural and electronic elements. Furthermore, optimising the injection moulding and extrusion processes for kenaf-based composite pellets could enhance the scalability of these materials for mass production. Limited studies have investigated the combined effects of printing parameters, fibre degradation and long-term environmental ageing on the mechanical reliability of 3D-printed kenaf composites for UAV applications.

### 4.2. Kenaf-Derived Carbon Materials for UAV Energy Storage Systems

One promising direction for advancing sustainable UAV technologies is the utilisation of kenaf-derived carbon materials in energy-storage systems. The high cellulose and lignin contents of kenaf biomass make it a suitable precursor to produce activated carbon, hard carbon and other porous carbon materials through pyrolysis, carbonisation and activation processes. These carbon materials possess tuneable pore structures, large specific surface areas and favourable electrochemical characteristics, making them attractive candidates for next-generation energy-storage devices. Although energy density remains the primary limitation of electric UAVs, current research on kenaf-derived carbon materials has largely focused on laboratory-scale supercapacitors and battery electrodes. Their integration into practical UAV energy-storage systems has not yet been demonstrated, representing a significant research gap.

Recent studies have demonstrated the successful conversion of KFs, core and biomass residues into carbon-based electrodes for supercapacitors and rechargeable batteries. Through chemical activation and surface engineering, kenaf-derived carbon materials have exhibited enhanced charge storage capabilities, high electrical conductivity and improved ion transport characteristics. Jung et al. [126] converted kenaf biomass into hard carbon anodes for sodium-ion batteries through acid pretreatment and controlled carbonisation, achieving a high initial Coulombic efficiency of 91.2% and enhanced Na^+^ diffusion kinetics owing to improved graphitic ordering and closed-pore structures. Furthermore, fluorine doping created a NaF-rich interphase that improved moisture resistance and interfacial stability, enabling the kenaf-derived anode to retain 87.3% of its capacity after 500 cycles under 500 ppm moisture.

For UAV applications, energy storage remains one of the most critical limitations affecting flight endurance, payload capacity and operational efficiency. The integration of kenaf-derived carbon materials into battery and supercapacitor systems may contribute to the development of more sustainable and lightweight energy-storage solutions [127]. Hong et al. [128] developed a kenaf stem-derived micro/mesoporous carbon as a sulphur host for lithium–sulphur batteries and achieved a high sulphur loading of 68 wt.% through effective sulphur encapsulation within the porous carbon structure. The polyvinylpyrrolidone-coated cathode exhibited a high specific capacity of 1228 mAh g^−1^ at 0.3 C-rate and maintained a reversible capacity of 632 mAh g^−1^ after 100 cycles at 0.3 C-rate, demonstrating the potential of kenaf-derived carbon materials as sustainable, high-performance energy-storage materials for future power systems. Supercapacitors fabricated from kenaf-derived activated carbon could provide rapid charge–discharge capabilities for peak power demands during take-off, landing and aggressive manoeuvres [129], while rechargeable battery systems could support extended flight operations.

**Figure 6:**
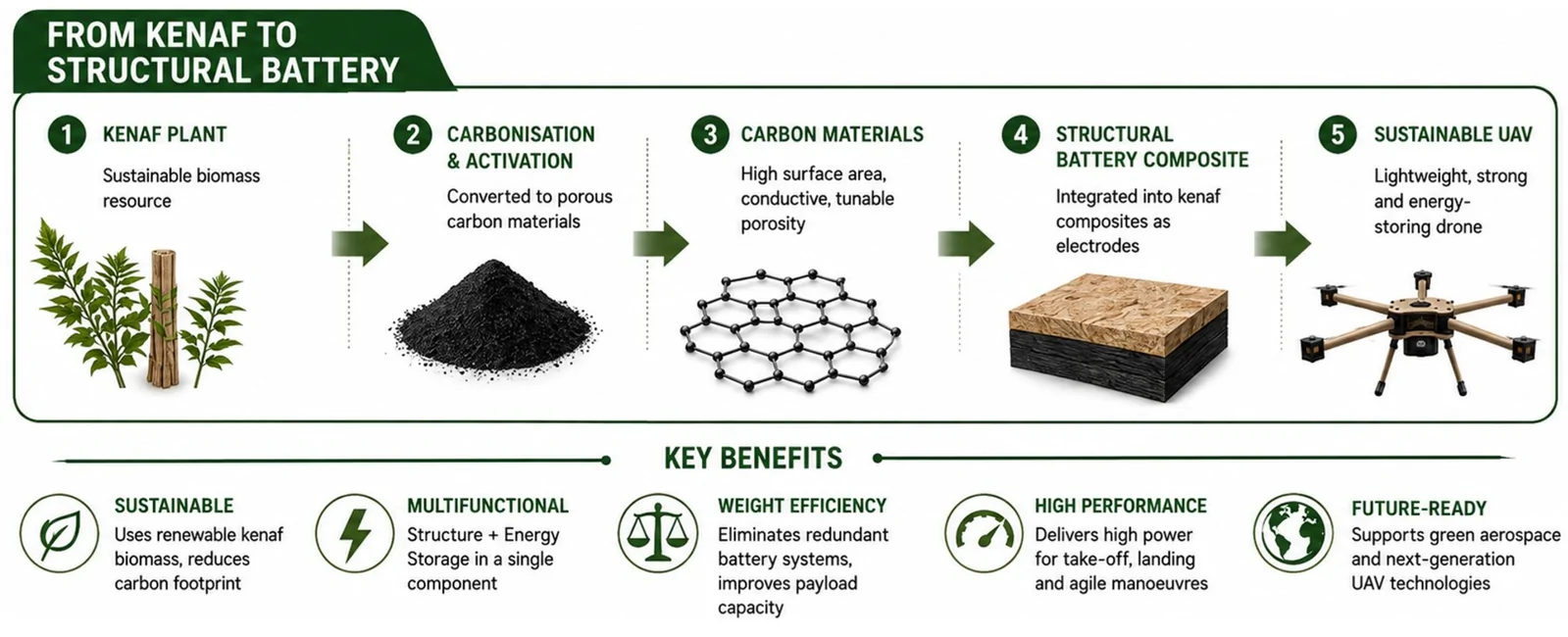
Conceptual workflow for the development of a kenaf-derived structural battery for sustainable UAV applications.

Another emerging research area is the development of structural energy-storage systems, in which load-bearing composite structures simultaneously function as energy-storage devices [130]. As shown in Figure 6, KF-reinforced composite panels, UAV arms or fuselage components could potentially integrate energy-storage functionalities while maintaining their structural role. This multifunctional approach could reduce overall system weight and improve energy efficiency by eliminating the need for separate structural and energy-storage components [131]. Although research on kenaf-based structural batteries remains limited, advances in carbonised natural fibres, conductive polymer matrices and multifunctional composite architectures suggest promising future opportunities. Future investigations should therefore focus on optimising carbonisation conditions, activation techniques, electrode architectures and electrochemical performance to facilitate the integration of kenaf-derived energy-storage materials into next-generation sustainable UAV systems.

### 4.3. Functionalisation for Smart UAV Applications

Beyond structural applications, KNFRPs can be functionalised to develop intelligent, multi-functional UAV components. These enhancements enable UAVs to monitor their own structural health, interact with their environment and even harvest energy during flight, expanding the operational capabilities of kenaf-based materials. One promising avenue is the integration of energy harvesting mechanisms into kenaf composites. By embedding piezoelectric materials such as barium titanate or zinc oxide nanoparticles into the kenaf matrix, UAV structures can convert vibrations or aerodynamic forces into electrical energy. This energy can power embedded sensors, extending battery life and enabling longer missions without additional power sources. Such self-sustaining systems are especially useful in surveillance, remote sensing and environmental monitoring UAVs.

Another promising functionalisation strategy involves the development of conductive polymer matrices for kenaf composites. Conventional thermoset and thermoplastic matrices used in KNFRPs are electrically insulating, limiting their ability to support sensing, signal transmission and electromagnetic shielding functions. By incorporating conductive polymers such as polyacetylene, polypyrrole, polyaniline, and polythiophene, kenaf composites can be transformed into multifunctional materials capable of electrical conductivity while maintaining lightweight structural performance. Such conductive matrices could enable integrated sensing networks, structural health monitoring systems and electromagnetic interference shielding within UAV airframes, reducing the need for separate electronic components and wiring. The incorporation of conductive nanomaterials like carbon nanotubes or graphene into kenaf composites can provide electromagnetic interference shielding and enable lightweight conductive pathways. These features are critical for UAVs operating in electronically noisy environments or for those requiring integrated circuits within the airframe.

Conductive kenaf composites also open possibilities for simplified PCB layouts, sensor arrays and signal routing directly within structural components. In line with sustainability goals, kenaf composites can be engineered to support printed sensors for monitoring environmental conditions such as humidity, strain and temperature. These sensors can be integrated during composite fabrication or printed onto kenaf-based films and boards. When applied to UAVs in agriculture or environmental research, these smart sensors provide real-time data while ensuring the system remains biodegradable and lightweight. Besides that, these functional composites also hold potential in lightweight communication systems. The biodegradable nature of kenaf-based boards and films makes them ideal substrates for printed antennas and RFID tags. These can be used for short-range telemetry, tracking or data transmission in disposable UAV systems, particularly for agriculture, disaster response or logistics. Incorporating such features would reduce reliance on conventional materials while enabling affordable and eco-friendly deployment of UAV fleets.

**Figure 7:**
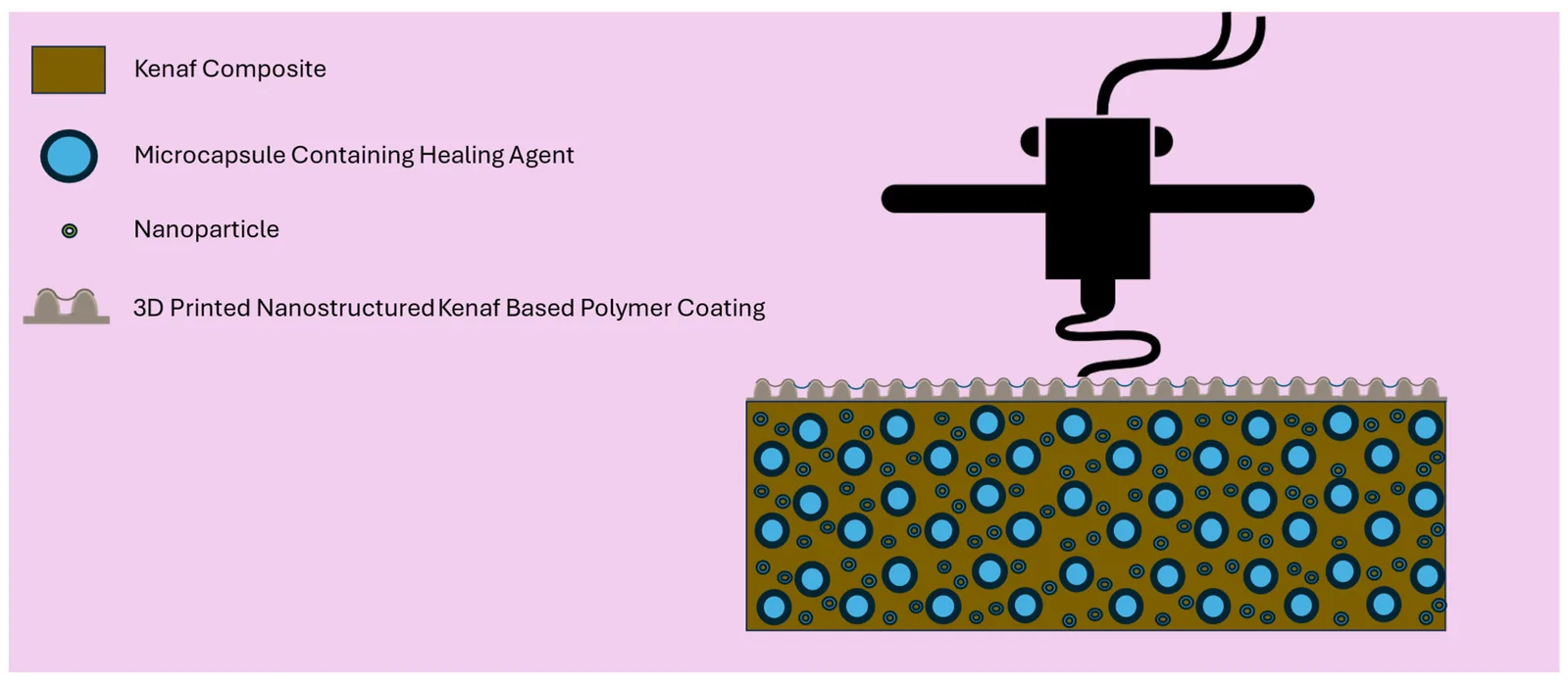
Multifunctional composite.

To further enhance durability and reduce maintenance costs, researchers are exploring self-healing composites. By embedding microencapsulated healing agents or shape-memory polymers within the kenaf matrix, small cracks and damage sustained during operation can be autonomously repaired. This functionalisation improves the reliability and lifespan of UAVs, particularly in demanding environments. Figure 7 serves as an example of such a multifunctional composite, showcasing a kenaf-based system designed with self-healing and enhanced performance features. It depicts a kenaf composite matrix embedded with microcapsules containing healing agents and dispersed nanoparticles for mechanical reinforcement. A 3D-printed nanostructured kenaf-based polymer coating is applied to the surface, enhancing its environmental resistance and functional integration. This combination of self-repair, strength and surface engineering exemplifies how bio-based materials like kenaf can be transformed into smart, multifunctional composites for high-performance applications. Together, these functionalisation strategies position KF composites not only as sustainable structural materials but also as enablers of smart, responsive and environmentally conscious UAV systems. Hence, continued research in nanomaterial integration, printed electronics and smart composite design will be essential to unlocking the full potential of kenaf in next-generation aerospace technologies.

### 4.4. Artificial Intelligence and Digital Material Design for Kenaf-Based UAV Systems

The development of kenaf-based composites for UAV applications can be significantly accelerated through the integration of artificial intelligence (AI), machine learning (ML) and digital material design approaches. Conventional material development often relies on extensive experimental trial-and-error processes, which are time-consuming, labour-intensive and costly. AI-driven methodologies offer opportunities to establish predictive relationships between fibre characteristics, processing parameters, environmental conditions and composite performance, thereby reducing the dependence on large-scale experimental campaigns.

Recent advances in ML have demonstrated considerable potential in predicting the mechanical, thermal and durability properties of composite materials based on material composition and manufacturing variables. For kenaf-based composites, AI models could be trained using datasets containing information on fibre treatment methods, fibre loading, fibre orientation, matrix selection, processing conditions and environmental ageing behaviour [132]. Such models may enable rapid prediction of tensile strength, flexural strength, impact resistance, thermal stability, moisture absorption and long-term durability under different service conditions. Arunachalam et al. [133] demonstrated the application of artificial neural networks (ANN) integrated with central composite design to predict and optimise the tensile strength of potassium permanganate-TKF/nanographene hybrid nanocomposites. The ANN model achieved an excellent prediction accuracy with an R^2^ value of 0.9805, accurately predicting an optimum tensile strength of 46 MPa compared with the experimentally validated value of 45.4 MPa, highlighting the potential of AI-driven approaches to accelerate material design and reduce the time and cost associated with composite development.

AI can also support the structural design and optimisation of UAV components fabricated from kenaf composites [134,135]. By integrating ML algorithms with FEA and multi-scale modelling, researchers can rapidly evaluate design alternatives and identify lightweight structures that satisfy specific strength, stiffness, vibration and impact resistance requirements. Such approaches are particularly valuable for UAV arms, fuselage shells, landing gear and internal support structures, where structural efficiency directly influences flight performance and payload capacity. Sadik et al. [136] developed an integrated framework combining experiments, FEA and explainable ML to predict the mechanical properties of natural fibre-reinforced hybrid composites. Among the evaluated models, XGBoost achieved the highest predictive accuracy, with R^2^ values of 0.71 for tensile strength and 0.76 for flexural strength, while SHapley Additive exPlanations analysis identified fibre length and filler content as the most influential parameters, demonstrating the potential of explainable AI to accelerate composite optimisation and reduce experimental trial-and-error.

Digital material design frameworks can further support the optimisation of manufacturing processes for kenaf-based UAV components [137,138]. For instance, in AM, parameters such as printing temperature, layer height, nozzle diameter, infill density and fibre content can be systematically optimised using data-driven approaches to improve interlayer bonding, dimensional accuracy and mechanical performance [138]. These predictive capabilities may significantly reduce material waste and development time while improving manufacturing consistency. Despite these opportunities, the successful implementation of AI-driven material design requires the establishment of comprehensive and standardised databases containing mechanical, thermal, environmental and manufacturing data for kenaf-based composites. Future research should therefore focus on developing open-access material databases, digital twins and hybrid physics-informed ML models to accelerate the qualification and deployment of kenaf composites in next-generation sustainable UAV systems.

### 4.5. Policy, Industrial Adoption and Market Readiness

The successful integration of kenaf-based composites into the UAV industry requires strategic policy interventions, industrial readiness and market adaptation. Establishing standardised testing and certification protocols is essential to ensure these materials comply with aerospace regulatory requirements. Regulatory bodies, alongside industry stakeholders, should collaborate on defining performance benchmarks and safety standards for BCs in UAV applications. KFs exhibit inherent variability in dimensions, chemical composition and mechanical properties due to differences in cultivation conditions, harvesting periods and climatic factors. This variability presents significant challenges in achieving consistent quality control and reproducibility during the large-scale manufacturing of kenaf-based UAV structures compared with synthetic fibre composites. Therefore, the establishment of standardised specifications and testing protocols for kenaf-based biocomposites is essential to ensure reliable material evaluation, facilitate certification processes and support their wider adoption in aerospace applications.

In addition to standardised testing, advanced predictive approaches such as multi-scale analysis can provide deeper insights into the relationship between material structure and performance. Stukhlyak et al. [139] demonstrated that multi-scale analysis enables the correlation of composite characteristics across different structural levels, allowing improved prediction and generalisation of material properties. A similar framework would be highly beneficial for kenaf-based biocomposites, where the hierarchical fibre structure, fibre–matrix interfacial interactions, defects and composite architecture collectively influence mechanical and environmental performance. By integrating microstructural characterisation, micromechanical modelling and macro-scale structural analysis, future research can develop more reliable predictive models for kenaf composites, accelerating their optimisation and qualification for advanced applications such as UAV structures.

To facilitate widespread adoption, partnerships between academia, industry and government agencies must be strengthened to bridge the gap between research innovations and commercial applications. Investment in pilot-scale production facilities and commercialisation pathways will be crucial to scale up manufacturing processes and enhance supply chain efficiency. Moreover, financial incentives, such as carbon credits, tax benefits and subsidies for sustainable materials, can accelerate industry-wide transition toward BC-based UAVs. According to Vayabari et al. [140], research on in vitro propagation methods (solid culture and liquid culture cultivation) for kenaf is crucial for sustainable cultivation, offering a cost-effective, labour-efficient and land-independent solution that supports economic growth, job creation and food security while contributing to Sustainable Development Goal 15 [141].

Market readiness also depends on comprehensive LCA studies that compare the environmental footprint of kenaf-based UAV components with traditional materials like carbon fibre and aluminium [142]. These assessments should evaluate factors such as energy consumption, greenhouse gas emissions and end-of-life disposal strategies [143,144,145]. A cradle-to-gate LCA study on PLA-based green composites filled with KF indicates that kenaf significantly reduces greenhouse gas emissions (up to 48%) and primary energy demand (up to 32%) compared to pure PLA [146]. To improve recyclability, future research should explore chemical and mechanical recycling methods, including solvolysis, pyrolysis and fibre–matrix separation techniques, ensuring the viability of circular economy principles in aerospace engineering. By addressing these challenges, kenaf-based composites can transition from experimental materials to mainstream UAV solutions, fostering a more sustainable and competitive market landscape.

## 5. Conclusions

KF has emerged as a promising material in the quest for sustainable solutions in high-performance industries, particularly in the development of UAVs. Its exceptional properties, including lightweight structure, impressive tensile strength and biodegradability, position it as an eco-friendly alternative to synthetic fibres like carbon and glass. This review highlights the significant potential of KFs to balance mechanical performance with sustainability, making them ideal for next-generation UAV components.

The integration of kenaf composites in advanced manufacturing processes, such as AM and injection moulding, further reinforces their role in enabling innovative and efficient designs. Looking forward, the future scope of KFs in UAV applications includes the development of hybrid composites that combine kenaf with high-performance fibres to enhance strength and durability, making them viable for more demanding structural roles. Nanotechnology-enabled enhancements, such as the incorporation of graphene or zinc oxide nanoparticles, are poised to improve thermal stability, mechanical strength, and moisture resistance. Moreover, kenaf-based materials show promise in smart UAV systems, where they could be functionalised for energy harvesting, embedded sensing, or self-healing capabilities.

Research into biodegradable and recyclable resin systems will further boost the environmental credentials of kenaf composites, supporting circular economy practices in aerospace. Finally, policy support, standardisation and industrial-scale processing will be critical in transforming kenaf from a niche material into a mainstream, high-performance solution for sustainable aviation.

## Figures and Tables

**Table 1:** Companies specialising in natural fibre composites worldwide.

Company	Country	Biocomposites	Properties	Applications
Amorim Cork Solutions [31]	Portugal	Cork composite	Acoustic insulation, thermal insulation, fire-resistant, lightweight, and hypoallergenic	Insulation, flooring, construction, fashion, design, health, energy production and aerospace
Arris Composites [32]	USA	Flax fibre-reinforced composite with additive moulding	High strength, high stiffness, lightweight and low-waste manufacturing	Bicycle spokes, performance footwear, aerospace, automotive, consumer electronics, drone and industrial
Bcomp [33]	Switzerland	Flax fibre-reinforced composite using ampliTex™ and powerRibs™	Lower CO_2_ footprint, lightweight, plastic reduction and natural damping	Automotive (interiors and body parts), sports equipment, aerospace, marine, consumer products
FlexForm Technologies [34]	USA	Natural fibre composite mats and panel products	Recyclable, mouldable, high strength, lightweight, mouldable and sound absorbent	Automotive, office interiors, aircraft, recreational vehicles, truck, commercial vehicles, industrial, modular housing and packaging
Greenboats [35]	Germany	Flax and cork composites	High strength, lightweight, and natural aesthetics	Sail boats, kayaks, motorboats, surf boards
HempFlax [36]	Netherlands	Hemp fibres and shives	Lightweight, durable, thermally stable	Construction, automotive panels, insulation, animal bedding
Jelu Werk [37]	Germany	Cellulose, wood fibre, wood plastic composites	Compostable, durable, and safe for food contact, cost-efficient	Food packaging, pet food, furniture, decking, noise protection walls and technical parts
Meshlin Composites [38]	Hungary	Flax, hemp, natural fibre composites	Flame retardant, impact-resistant and eco-friendly	Wall panels, furniture, E-vehicles, sporting goods
Midwest Composites [39]	Malaysia	Kenaf, hemp, flax, jute, palm oil fibres and hybrid natural fibre composites (Glass/Kenaf)	Lightweight, high strength-to-weight ratio	UAV fuselages, wings and flaps, agricultural drone components, bus engine covers, automotive body panels, furniture and mass transit interior components
Natural Fibre Technologies [40]	Canada	Hemp bast fibre and hurd fibre composites	Lightweight, highly absorbent, dust-free and biodegradable	Commercial manufacturing, garden mulch, animal bedding, architectural millwork and acoustic dampening
Polyvlies [41]	Germany	Natural fibre composites (flax, hemp, kenaf, jute, sisal, wool and cotton)	Sustainable, natural fibre blend and customisable properties	Automotive, furniture, roof system components, insulation materials, mulch
Suxus [42]	Malaysia	Kenaf, pineapple leaf	Lightweight, durable, sustainable, and high strength-to-weight ratio	UAVs, quadcopters, educational drones, pultruded profiles
Tecnaro [43]	Germany	Wood, lignin-based composites (e.g., Arboblend^®^, Arboform^®^)	High rigidity, low shrinkage, excellent acoustic properties, moderate to high thermal resistance, natural odour or odourless, moderate to high impact strength, scratch resistance and flame-retardant	Construction, landscaping, agriculture, technical parts, furniture, toys, sport and leisure, household items, musical instruments, stationery items, clothing industry, packaging
Trex [44]	USA	Wood (recycled wood fibres and plastic)	Weather-resistant, low maintenance, insect/rot-resistant, and UV resistant	Outdoor decking, railing, fencing, cladding, furniture
Trifilon [45]	Sweeden	Natural fibre BCs	High performance, low CO_2_, lightweight	Automotive panels, consumer products, outdoor gear

**Table 2:** Past research on KFRC and hybrid KFRC.

Year	Ref.	Materials	Variable Parameter	Best Parameter	Analysis	Results
KFRC						
2023	Hayeemasae et al. [53]	Kenaf/recycled high density polyethylene/natural rubber	KF loading (0, 10, 20, 30 and 40 phr ^1^)	10 phr ^1^	Tensile strength after 12 months	Increased the retention properties by 25%
					Elongation at break after 12 months	Increased the retention properties by 5%
2022	Setyayunita et al. [54]	Kenaf/epoxy	NaCl in the treatment solution (1 wt.%, 3 wt.% and 5 wt.%) and the duration of immersion of fibres in the solution (1 h, 2 h and 3 h)	5 wt.% NaCl	Flexural strength	2.085 GPa
					Flexural modulus	19.77 MPa
					Internal bonding	1.8 MPa
					Thickness swelling	3%
					Water absorption	10.9%
2020	Azammi et al. [55]	Kenaf filled natural rubber/thermoplastic polyurethane	Proportions of kenaf, NR ^2^ and TPU ^2^	Kenaf 12.5% NR ^2^ 50% TPU ^2^ 37.5%	Water absorption	<1%
					Density	1.03 g/cm^3^
					Thickness swelling	4%
					Damping factor	tan *δ* > 0.3
Hybrid KRFC						
2024	Jothiprakash et al. [56]	Pineapple leaf fibre/KF/vinyl ester	Ratio of PALF:KF:VE ^2^ (0PALF/0KF/100VE, 8PALF/8KF/84VE, 12PALF/12KF/76VE, 16PALF/16KF/68VE, 20PALF/20KF/60VE and 24PALF/24KF/52VE)	20PALF/20KF/60VE ^2^	Tensile strength	44 MPa
					Flexural strength	43 MPa
					Compressive strength	114 MPa
					Impact strength	58.9 kJ/m^2^
					Microhardness	39.5 HV
					Specific wear rate	9.7 × 10^5^ mm^3^/nm
					Coefficient of friction	0.213
2023	Afolabi et al. [57]	Core syntactic foam composite (SFC) reinforced with face-sheets of combined kenaf and glass fibres	Stacking sequences of face-sheet reinforcement (kenaf-kenaf, glass-glass, glass-kenaf and kenaf-glass)	Glass-kenaf	Tensile strength	40.39 ± 4.81 MPa
					Tensile modulus	2.14 ± 0.315 GPa
					Flexural strength	159.51 ± 26.18 MPa
					Flexural modulus	7.16 ± 0.68 GPa
				Kenaf-glass	Compressive strength	63.81 ± 3.67 MPa
					Compressive modulus	1.65 ± 0.26 GPa
2022	Azlin et al. [58]	Kenaf/polyester fibre/PLA	Laminate configuration	S5 (K/K/P/K/K) ^2^	Glass transition temperature (*T_g_*)	58.90 °C
					Melting temperature (*T_m_*) Burning rate	95.49 °C
						29 mm/min
2021	Loganathan et al. [59]	*Cyrtostachys renda*/kenaf/multi-walled carbon nanotubes/phenolic	Different ratios of *Cyrtostachys renda* and kenaf reinforced with 0.5 wt.% MWCNT-phenolic composites (10C:0K, 7C:3K, 5C:5K, 3C:7K, 0C:10K) ^2^	5C:5K ^2^	Tensile strength	47.96 MPa
					Flexural strength	84.61 MPa
					Impact strength	7.28 kJ/m^2^
					Water absorption	26.93%

^1^ phr denotes parts per hundred rubbers; ^2^ C denotes *Cyrtostachys renda*, K denotes kenaf, MWCNTs denotes multi-walled carbon nano-tubes, NR denotes natural rubber, PALF denotes pineapple leaf fibre, TPU denotes thermoplastic polyurethane, VE denotes vinyl ester.

**Table 3:** Research to date on KNFRP palletisation techniques.

Year	Ref.	Materials	Fabrication Technique	Variable Parameter	Best Parameter	Analysis	Results
2024	Zaki et al. [60]	Kenaf/PP/malleated polypropylene	Extrusion and injection moulding	Addition of MAPP ^2^ as coupling agent (0 g, 5 g, 10 g)	K/PP/MAPP ^2^ (10 g/180 g/10 g)	Tensile strength	29.359 MPa
						Impact strength	1971.994 J/m^2^
						Water absorption	1.05%
2024	Khan et al. [61]	Bamboo/kenaf core/PLA	Extrusion and compression moulding	Bamboo to kenaf core weight ratio	BF-PLA ^2^	Tensile strength	25.95 MPa
						Compressive strength	173.15 MPa
					BF30-KF70	Impact strength	6.6 Nm
					KF-PLA	Hardness resistance	69.2 HRS
					BF30-KF70	Thermal degradation temperature	484 °C
2023	Tamta and Palsule [62]	KF/chemically functionalised styrene acrylonitrile	Extrusion and injection moulding	Fibre loading	30/70 KF/CF-SAN ^2^	Tensile strength	78% increment
						Tensile modulus	75% increment
						Flexural strength	125% increment
						Flexural modulus	104% increment
2023	Hassan et al. [63]	PLA/recycled high density PE/kenaf foamed with exothermic azodicarbonamide	Extrusion and compression moulding	Composition of PLA, rHDPE ^2^ and ADC ^2^ foam	3 phr ^1^ ADC-foamed PLA/rHDPE/KF ^2^ (90/10/30)	Flexural strength	6.61 MPa
						Flexural modulus	3052.22 MPa
						Temperature at 10% weight loss	300.9 °C
						Burnt residue	9.4%
2021	Woo and Cho [64]	Chopped kenaf/PLA/ammonium polyphosphate	Extrusion and compression moulding	APP ^2^ Loading	50 APP-PLA ^2^	Flammability Test	89% increment
						Tensile Modulus	32% increment
						Storage modulus	165% increment
2020	Mustapa et al. [65]	Kenaf Core/PP	Extrusion and compression moulding	Fibre Loading (5, 10, 15, 20 and 25 wt.%)	20 wt.% kenaf core	Flexural Strength	48.37 MPa
						Flexural Modulus	1.76 GPa
2020	Azam et al. [66]	Multiwalled carbon nanotubes/short kenaf (20 mesh)/PP	Injection moulding	Fibre loading (10 to 40 wt.%) and MWCNT ^2^ loadings (1 to 4 wt.%)	PP 30 K	Water absorption	8%
					PP/30K/3MCT	Flammability Rate	11 mm/min
2020	Hassan et al. [67]	KF/PLA/azodicarbonamide foaming agent	Extrusion and hot cold press moulding	Effects of two extrusion temperature profiles (P1:175, 180, 185, 170 °C and P2: 165, 170, 175, 160 °C) and the loadings of ADC ^2^ (1–5 phr ^1^)	P2 (165, 170, 175, 160) °C and ADC ^2^ loading at 3 phr ^1^	Tensile strength	~5 MPa
						Moisture content	2.8%
						Water absorption	10%
						Onset temperature (*T_o_*)	265 °C
						Peak decomposition temperature (*T_peak_*)	312 °C
						Thermal residue	6.7%

^1^ phr denotes parts per hundred rubber; ^2^ BF denotes bamboo fibre, K denotes kenaf, ADC denotes azodicarbonamide, APP denotes ammonium polyphosphate, CF-SAN denotes chemically functionalised styrene acrylonitrile, MAPP denotes malleated polypropylene, MWCNTs denotes multi-walled carbon nano-tubes, rHDPE denotes recycled high density polyethylene.

**Table 4:** Research to date on AM of kenaf composites.

Year	Ref.	Materials	Variable Parameter	Best Parameter	Analysis	Results
2023	Alaa et al. [70]	10 wt.% kenaf/PLA	Untreated and treated KFs using superheated steam	Untreated kenaf	Tensile	27.27 MPa
				Treated kenaf	Dynamic mechanical analysis	222.15 MPa
				Treated Kenaf	Water absorption	14.81%
2023	Hamat et al. [71]	Self-polymerised polydopamine coating on kenaf/PLA	Fibre loading (5, 10, 15 and 20 wt.%)	5 wt.%	Tensile strength	54.6 MPa
				5 wt.%	Flexural strength	75.2 MPa
				20 wt.%	Compressive strength	108.6 MPa
2023	Lau et al. [72]	Kenaf/PLA	Fibre loading (0, 5, 10, 15 and 20 wt.%)	15 wt.%	Tensile strength	47.02 MPa
					Tensile modulus	65.95 GPa
					Max strain	3.71%
					Yield stress	1.04 MPa
2022	Shahar et al. [73]	Kenaf/PLA	Fibre loading (3, 5 and 7 wt.%)	3wt.%	Tensile	54.78 MPa
				3wt.%	Impact	3.19 kJ/m^2^
				7wt.%	Fatigue	Longest fatigue life and good load-carrying capacity in the high cycle region
2022	Khalid et al. [74]	Kenaf/PP	NaOH concentration for fibre treatment (2%, 4%, 6%, 8% and untreated)	6%	Tensile strength	267.69 MPa
					Tensile Modulus	11.88 GPa
					Fracture strain	2.07%
			Injection temperature (190, 200 and 210 °C)	200 °C	Shear modulus	769.3 MPa
					Average fibre orientation	0.9818
2021	Aumnate et al. [75]	Kenaf Cellulose/PLA	Tetraethyl orthosilicate surface treatment and polyethylene glycol 6000 addition (PLA/KFs, PLA/PEG4/KFs and PLA/PEG6/KFs) ^1^	PLA/PEG6/KFs ^1^	Tensile	Around 45 MPa
					Glass transition temperature (*T_g_*)	57.6 °C
					Water contact angle	81.1° ± 6.2°

^1^ PEG denotes polyethylene glycol.

**Table 5:** Past research on KPP, KCB, KFM and KBF.

Year	Ref.	Materials	Variable Parameter	Best Parameter	Analysis	Results
KPP						
2024	Hashim et al. [79]	KPP	KPP and Kraft pressed paper	KPP	Tensile strength	48 MPa (8% reduction compared to Kraft pressed paper)
					Thermal stability	345 °C (2.87% higher than Kraft pressed paper)
					Relative permittivity at 50 Hz	2.41
2023	Umair et al. [80]	KPP/polyvinyl alcohol	Concentration of PVA ^2^ (0, 2, 4 and 6%) ^1^	6% PVA ^2^ weight percentage concentration	Tensile index	81.36 Nm/g
					Burst index	6.73 kPam^2^/g
					Tear index	5.89 mNm^2^/g
					AC breakdown voltage	36.30 kV
					AC breakdown Strength	157.76 kV/mm
2021	Adnan et al. [81]	Empty fruit bunch/KPP	EFB ^2^ to kenaf ratio (0:100, 75:25, 50:50, 25:75, 100:0)	100% kenaf	Tensile strength	65.88 MPa
					Lightning impulse Breakdown voltage	31.47 kV/mm
					AC breakdown voltage	47.77
KCB						
2022	Asri et. al. [82]	Kenaf particle/phenol formaldehyde adhesive	Resin content in phenol formaldehyde adhesive (8 &10%) and 2% NaOH treatment	10% resin, 2% NaOH treated	Flexural strength	3.35 MPa
					Flexural modulus	955.82 MPa
					Internal bonding strength	0.16 MPa
					Thickness swelling	57.28%
					Water absorption	144.21%
2011	Zawawi et al. [83]	Kenaf/PLA and rice-husk/PLA	Fibre type (Kenaf and rice-husk)	Kenaf/PLA	Permittivity (ε’)	3.3 down to 2.5 (higher than rice-husk)
					Loss tangent (tan δ)	low loss tangent not exceeding 0.1 (similar to rice-husk)
KFM						
2025	Chong et al. [84]	Lightweight foamed composite/0.6% treated kenaf fibre (TKF)	LFC ^2^ with and without KFs	LFC-TKF 0.6 ^2^	Compressive strength	4% increment
					Flexural strength	56% increment
					Tensile strength	62% increment
2023	Afolabi et al. [85]	SFC reinforced with face-sheets of combined kenaf and glass fibres	Sequences of face-sheet reinforcement (kenaf-kenaf; glass-glass; glass-kenaf and kenaf-glass)	Glass-kenaf	Hardness	49.8 Hv
					Water absorption after 720 h	2.118% (65.6% lower compared to KK)
					Lowest buoyancy force	0.250 ± 0.34 N
				Kenaf-glass	Lowest sound level (dB)	52.06 ± 4.79
					Lowest sound pressure (dB)	128.58 ± 0.61
2023	Samaei et al. [86]	Kenaf/polyurethane	KF loading and length	KF loading of 1.2 wt.% and length of 8 mm	Sound absorption average	0.65 (35.41% higher than neat PU ^2^)
2023	Zhang et al. [87]	TKF/geopolymer-foam fibre	Addition of TKF and its waste liquid	TKF/GFF ^2^	Thermal Conductivity	0.118–0.248 W/(m·k)
					Compressive strength	1.6–19.9 MPa
2022	Siagian et al. [88]	Cellulose nano-fibrils extracted from East Java, Indonesia based KFs/PU ^2^	CNF ^1^ loading (0, 3, 5, 7 and 10 wt.%)	3 wt.% CNF/PU ^2^	Compressive strength	284.434 kPa (20.01% increment)
					Compressive modulus	7.32 MPa (29.21% increment)
					Flexural strength	734.15 kPa (28.29% increment)
2020	Soberi et al. [89]	Kenaf core/polyurethane foam/expandable graphite/aluminium hydroxide	Additive loading EG ^2^ (5, 10, 15 wt.%) ATH ^2^ (0, 2, 4, 6 wt.%)	PU/KF/6ATH/10EG ^2^	Compressive strength	~0.2 MPa
					Compressive modulus	~5 MPa
2020	Majid et al. [90]	Rigid palm oil-based polyurethane/KF	KF loading (2.5, 5.0, 7.5, 10.0 and 12.5 pphp ^1^)	7.5 phpp ^1^	Compressive strength	0.8963 MPa
KBF						
2022	Hazrol et al. [91]	Kenaf/Corn starch	Fibre loading (0, 2, 4, 6 and 8%)	0.6% KF Loading	Tensile strength	17.74 MPa
					Tensile modulus	1324.74 MPa
					Elongation at break	48.79%
					Moisture content	33.67%
					Water solubility	5.99 ± 2%
					Crystallinity index	45.06%
2021	Oyekanmi et al. [92]	Cellulose nanofibrils isolated from kenaf bast fibres/macroalgae	Fibre loading (1, 2, 3, 4 and 5%) and silane treatment	Silane treated, 4% fibre loading	Tensile strength	37.5 MPa
					Tensile modulus	Around 300 MPa
					Elongation at break	Around 42%
					Toughness	Around 4 N
					Water contact angle	91.8° (high hydrophobicity)

^1^ pphp denotes part per hundred polyols; ^2^ ATH denotes aluminium hydroxide, CNF denotes cellulose nano-fibrils, EFB denotes oil palm empty fruit bunch, EG denotes expandable graphite, GFF denotes geopolymer-foam fibre, LFC denotes lightweight foamed composite, PU denotes polyurethane, PVA denotes polyvinyl alcohol.

## Data Availability

No new data were created or analyzed in this study. Data sharing is not applicable to this article.

## Abbreviations

The following abbreviations are used in this paper:

AFRP	Aramid fibre-reinforced polymer
AI	Artificial intelligence
AM	Additive manufacturing
ANN	Artificial neural networks
BC	Biocomposite
CFRP	Carbon fibre-reinforced polymer
FEA	Finite element analysis
FDM	Fused deposition modelling
GFRP	Glass fibre-reinforced polymer
KCB	Kenaf composite board
KBF	Kenaf biofilm
KF	Kenaf fibre
KFM	Kenaf foam
KFRC	Kenaf fibre-reinforced composite
KNFRP	Kenaf natural fibre-reinforced polymer
KPP	Kenaf Pressed Paper
LCA	Life cycle assessment
ML	Machine learning
PCB	Printed circuit board
PE	Polyethylene
PLA	Polylactic acid
PP	Polypropylene
SFC	Syntactic foam composite
TKF	Treated Kenaf Fibre
UAV	Unmanned aerial vehicle
UV	Ultraviolet
